# Advances in Microfluidics for Single Red Blood Cell Analysis

**DOI:** 10.3390/bios13010117

**Published:** 2023-01-09

**Authors:** Georgii V. Grigorev, Alexander V. Lebedev, Xiaohao Wang, Xiang Qian, George V. Maksimov, Liwei Lin

**Affiliations:** 1 Data Science and Information Technology Research Center, Tsinghua Berkeley Shenzhen Institute, Tsinghua University, Shenzhen 518055, China; 2 Mechanical Engineering Department, University of California in Berkeley, Berkeley, CA 94720, USA; 3 School of Information Technology, Cherepovets State University, 162600 Cherepovets, Russia; 4 Machine Building Department, Bauman Moscow State University, 105005 Moscow, Russia; 5 Shenzhen International Graduate School, Tsinghua University, Shenzhen 518055, China; 6 Faculty of Biology, Lomonosov Moscow State University, 119991 Moscow, Russia; 7 Physical metallurgy Department, Federal State Autonomous Educational Institution of Higher Education National Research Technological University “MISiS”, 119049 Moscow, Russia

**Keywords:** RBC, red blood cell, erythrocyte, single cell, microfluidics, medicine

## Abstract

The utilizations of microfluidic chips for single RBC (red blood cell) studies have attracted great interests in recent years to filter, trap, analyze, and release single erythrocytes for various applications. Researchers in this field have highlighted the vast potential in developing micro devices for industrial and academia usages, including lab-on-a-chip and organ-on-a-chip systems. This article critically reviews the current state-of-the-art and recent advances of microfluidics for single RBC analyses, including integrated sensors and microfluidic platforms for microscopic/tomographic/spectroscopic single RBC analyses, trapping arrays (including bifurcating channels), dielectrophoretic and agglutination/aggregation studies, as well as clinical implications covering cancer, sepsis, prenatal, and Sickle Cell diseases. Microfluidics based RBC microarrays, sorting/counting and trapping techniques (including acoustic, dielectrophoretic, hydrodynamic, magnetic, and optical techniques) are also reviewed. Lastly, organs on chips, multi-organ chips, and drug discovery involving single RBC are described. The limitations and drawbacks of each technology are addressed and future prospects are discussed.

## 1. Introduction

Normal human RBCs are biconcave-shaped discs of about eight µm in diameter and two µm in thickness, and they can undergo passive deformations while maintaining mechanical stability during the microcirculation process. Over the lifespan, the RBCs lose their membrane integrity and degrade by the mononuclear phagocytic system [1]. Understanding the dynamics of RBCs, specifically under confined studies, is an exciting area in biomechanics. Researchers have studied the complex interactions between RBC and deformable microchannels and their relationships with hydrodynamic flows. It is noted that the moving velocities of RBCs can affect the wall deformability in microchannels [2]. Furthermore, blood cell counts that comprise WBC, RBC, platelets, and hemoglobin can be utilized for multiple clinical diagnoses and measurements. Microfluidic biochips capable of remote and partial blood counts are in high demand. Researchers have attempted to build devices that can effectively characterize the blood-count techniques, including a dynamic range of cell counting, leukocytes, and three-part differentials [3].

**Figure 1:**
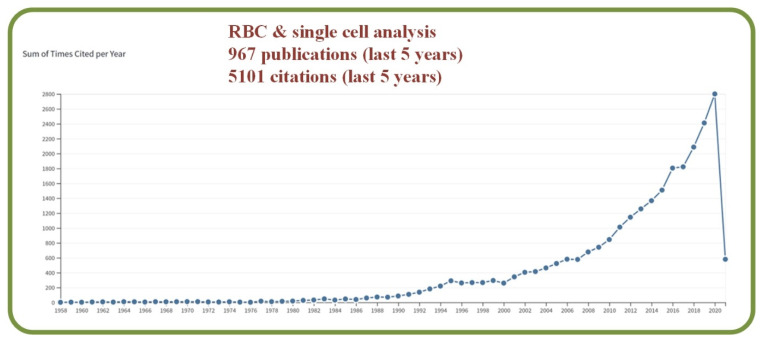
Graph created with the Clarivate Analytics citation report, with a topic search of “RBC” AND “single cell analysis”.

The study of living cells in artificially constructed environments has been a key research topic in the fields of pharmaceuticals, biology, healthcare, military, and others. Manz and his team are the early researchers to analyze fluidic flows at the microscale with the proposed key concept of Total Chemical Analysis (TAS) system [4]. With the increased capability of managing sub-microliter liquid volumes in predictable laminar flows, microfluidic devices can extract and deliver materials such as living cells with enhanced precisions. As such, single-cell assessments, live-cell imaging, organ-on-a-chip, transcription factor, binding assessments, and high throughput antibody screening procedures have all been investigated in recent years with the assistance of microfluidic devices [5]. Statistically, the Clarivate Analytics citation report with a topic search of “RBC” AND “Single cell analysis” show 967 publications with 5101 citations in the last 5 years as shown in Figure 1.

Specifically, microfluidic technologies have been widely reported in papers studying living cells, including cell counts, cell analysis, cell sorting, and the analysis of cell functioning or drug sensitivity in high-throughput screening processes [6]. While these versatile methods have been shown to be very effective in the manipulation of living cells, limitations are found in problems such as the characterizations of sickle cell disease, malaria infection, and sepsis [7] as well as other challenging issues [8,9].

While microfluidic technologies are helpful for the single-cell analysis, specific controls for efficient manipulations and analyses are required to have good repeatable cell growth, morphological factors, population heterogeneity, and characterizations [10]. Some studies have utilized the environmental controllability, cell input, intracellular traits, qualitative analytics, and integrated theories [11]. There have also been many microfluidics-based single-cell technologies such as: droplet-based, deterministic lateral displacement technology, hydrodynamic pressure-based manipulation, and microarray-based methods [12]. Microfluidic systems can also be used in studying the immune responses, including single immune cell analysis, genomics, proteomics, cell signaling, cell-to-cell, and cell-to-environment interactions [13]. In order to address the vast potential applications, different microfluidic platforms have been proposed, such as dielectrophoretic-based devices with 3D microelectrodes [14], enhanced single-cell sorting with fiber lasers [15], and systems with 3D microstructures [16]. The traditional micro-fabrication process suffers from multiple drawbacks, including limitations in the construction of 3D architectures, expensive and time-consuming device designs, and difficulties in mass productions from prototypes. 3D printing comes as an efficient alternative in mitigating such problems [17]. Milli-fluidics is a domain that focuses on 3D printing for biological and chemical analyses. Standing on a scale of above 200 μm, the key 3D fabrication methods include stereolithography, polyjet, or fused deposition modeling (FDM). Other 3D printing processes rely on methods such as transfer molding, extrusion-based 3D printing, 3D stereolithography, multijet modeling [18,19,20], binder jetting, laser sintering, laser melting, electron beam melting, and hybrid processes [21]. The microfluidic construct can also incorporate functional elements, including actuators (valves, pumps, multiplexers) and sensing elements via the 3D printing technology [22].

Further advances have enabled the integration of an on-chip processing system that assimilates dilution, lysis, and filtration capabilities. The proposed device also features subsystems for sample processing and electrical measurements in solutions of different viscosities with increased device performance characteristics [23]. Similarly, a proposed micro-gas exchanger embedded with a microfluidic platform can effectively assess the adhesion of red blood cells under hypoxic flows while mimicking the function of postcapillary venules [24]. This paper aims to provide the viability study of using single cell-based microfluidic technology for various applications with a focus on state-of-the-art RBC/erythrocyte research.

## 2. Integrated Sensors for Microfluidic Platforms for Assessing Single Erythrocytes

This section reviews previous works in analyzing the hemodynamics of Red Blood Cells (RBCs) or erythrocyte using microfluidic or Lab-on-a-chip (LOC) platforms. The primary tools include microscopic flow assessments, smartphone-based detections, multispectral imaging (UV-Vis/NIR/Raman), and tomographic analyses. Techniques such as speckle analysis, waveguide sensors, machine vision, and optical tweezers have also been reported.

### 2.1. Microscopic RBC Flow Analysis

**Figure 2:**
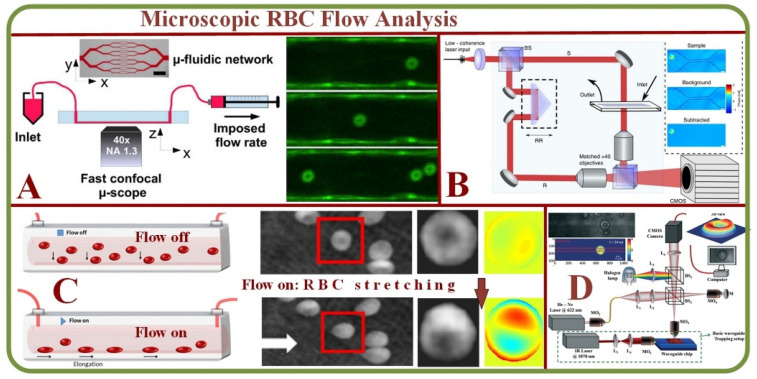
Microscopic RBC Flow Analyses. (**A**) Microvasculature on a chip: the study of RBCs, microfluidic network, and confocal images of RBCs in a channel [27]. (**B**) System for quantitative phase imaging of erythrocytes under microfluidic constriction in a high refractive index medium [28]. (**C**) Hydrodynamic RBC deformation by quantitative phase microscopy and Zernike polynomials: the principle, experimental results and simulation photos of RBC stretching under the fluidic flow and without the fluidic flow [29]. (**D**) Schematic diagram of the quantitative phase microscopy of RBCs during planar trapping and propulsion [30].

The microvasculature is a complex and dynamic system with blood constituents, primarily RBCs of about 40–45% by volume. Micro-scale blood flows have been efficiently analyzed in-vitro using the combination of microscopic imaging techniques (bright field or confocal) equipped with microparticle image- or tracking-velocimetry (µPIV or µPTV). Researchers have also examined hematocrit profiles and viscosity characteristics in bifurcating geometries using an inverted microscope above the microchannel [25]. The scheme of Quantitative Phase Imaging (QPI) offers high sensitivity but has restrictions in multiple cell imaging capabilities. Analyzing RBCs with this method provides a uniform refractive index as deduced from microfluidic channels [26]. Figure 2 shows several device examples for microscopic RBC flow analyses. Figure 2A is an example of microvasculature on a chip for the confocal microscopy study to characterize the endothelial cell properties and the near-wall motions of RBCs [27]. Figure 2B is a system for quantitative phase imaging of erythrocytes under microfluidic constriction in a high refractive index medium [28]. Figure 2C shows sketches and photos of the hydrodynamic RBC deformation by the quantitative phase microscopy and Zernike polynomials [29]. Figure 2D exhibits the diagram of the quantitative phase microscopy of RBCs during planar trapping and propulsion [30].

Specifically, QPI combined with waveguide trapping has been used to study alterations in the RBC morphology during planar trapping and transportation while simultaneously recording the changes in the time-lapsed images of trapped RBCs via interference microscopy to construct optical phased maps, while the hydrodynamic deformation of RBCs in a microfluidic channel via QPI has been conducted using the digital holography [25,27,29,30,31,32]. 3D Holographic Tomography being one of the most powerful 3D QPI methods advances further by combining various techniques into multimodal operations, integrating Raman imaging, Brillouin spectroscopy or fluorescence [33,34,35]. Artificial intelligence algorithms and machine learning approaches impact the system architecture improving measurement accuracy becoming the focus in 3D QPI systems [36,37,38,39,40,41,42].

On the other hand, Atomic Force Microscopy (AFM) has been reported to efficiently assess RBC stiffness and ability to move in microcapillaries after being exposed to intravenous fluid (IVF) [43,44] or high levels of oxidative stress [45].

### 2.2. Tomographic Analysis of Erythrocyte Flow

**Figure 3:**
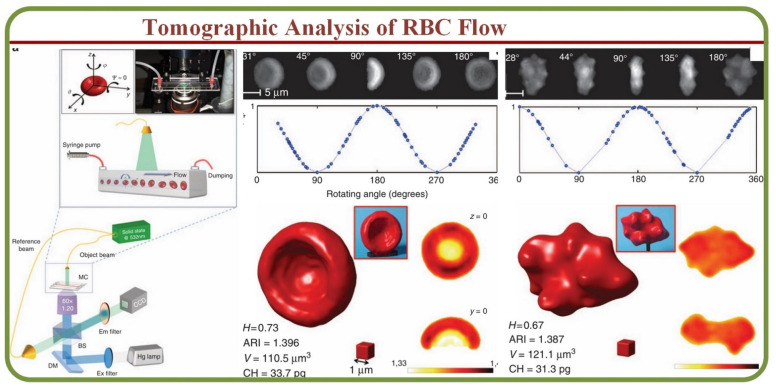
The tomographic analyses of RBC Flows. RBC tomographic flow cytometry by the digital holography. Healthy and morphological RBC anomaly phase images and 3D reconstructions [50].

The assessment and visualization of individual RBCs are crucial in many situations as the single-cell physiology can open pathways to understand interactions under multi-particle or suspension environments [46]. Modern techniques such as optical coherence tomography (OCTA) enable the visualization of functional networks non-invasively to detect in-vivo microvasculature [47]. Furthermore, label-free acoustic-based microfluidic platforms can effectively assess hydrodynamic junctions in microchannels to analyze individual cells [48]. Other prominent methods for the tomographic analysis include the full phase-contrast tomography and rotational erythrocyte aggregates [49] for flow cytometry applications [50]. For example, single RBC cytometry images by a digital holography setup is shown in Figure 3A, including the microfluidic chip, setup, healthy and morphological RBC anomaly phase images, and 3D reconstructions [50]. These tomography tools enable the label-free specific 3D tomography of biological samples through hyperspectral optical diffraction techniques [51].

### 2.3. Smartphone-Based Analysis of Single RBCs

**Figure 4:**
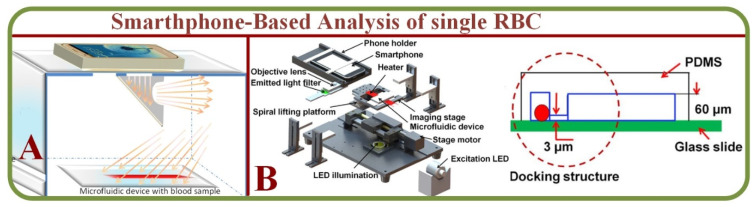
(**A**) A smartphone-based optical platform for the colorimetric analysis of different hematocrit samples without and with PDMS light diffuser and the corresponding gray scale values [52]. (**B**) A RBCs system capable of migration assay based on the microfluidic device and smartphone [54].

Microfluidics with smartphone-based applications have addressed issues associated with conventional microfluidic devices. For example, Kim and colleagues proposed smartphone-based optical platforms for the colorimetric analysis of blood hematocrit [52] as shown on Figure 4A. Disposable paper– and plastic-based microfluidic platforms are feasible for colorimetric analysis by incorporating paper-based conventional reagent test strips embedded inside plastic LOC microchannels. This device has successfully investigated a small volume of urine analytes for colorimetric analysis of glucose, protein, pH, and RBCs [53]. Microfluidic devices and smartphone technology have also been implemented to construct functional cell assays, primary RBC lysis to test cell migration properties as shown in Figure 4B [54]. This platform is effective for future cell migration and medical diagnostics research.

Smartphone-based applications for Point-of-care testing (POCT) is another emerging approach. Laypersons are able to identify and count cells through simple manipulation using “in-flow” imaging of 3 microL fingertip whole blood. With relatively high throughput reported (~8000 cells/min) with a 30-fold dilution ration of whole blood Zhang et al. showed their device ability to detect abnormal RBCs concentration in 75 cases of clinical patients, as well as RBC abnormal morphology [55]. Other examples of simple-to-fabricate, cost-efficient, and easy-to-use microfluidic “sample-in & answer-out” POCT devices were successfully tested on detecting plasma creatinine from finger-pricked blood by separation of induced by calcium ions aggregating RBCs from plasma ans testing it via capillary action with alkaline picrate reagent, resulting in a colorimetric signal. This smartphone-based anylyses has 2–3 min turn-around with 94% accuracy and the coefficient of variation ranging from 0.64 to 6.4% [56].

Ding et al. reported compact centrifugal microfluidic platform with smartphone-based image processing for parallel RBC antigen typing further improving the imaging technique by introducing machine learning algorithm and achieving 94.10% in the micro-weighted performance evaluation [57].

### 2.4. Spectroscopic Analysis of Single RBCs

**Figure 5:**
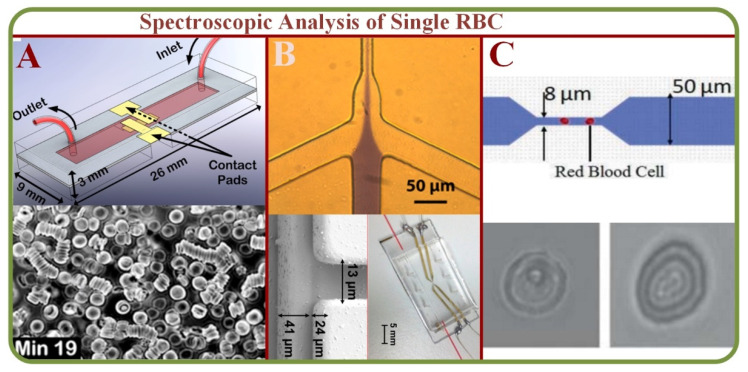
(**A**) Quantitative absorption imaging of RBCs for the determination of physical and mechanical properties [59]. (**B**) The optofluidic single RBC system for POC malaria diagnosis with images of healthy and infected RBCs [60]. (**C**) Microfluidic fluctuation spectroscopy for cellular viscoelastic measurement of RBCs and graphs [61,62].

The spectral imaging technique can integrate conventional images with spectroscopic methodologies to obtain spatial and spectral characteristics of an object. Spectral imaging systems are either multispectral, hyper-spectral, or ultra-spectral according to the biochemical components [58]. Studies indicate that RBCs’ physical and mechanical properties such as hemoglobin concentration, total volume, and membrane characteristics can be effectively analyzed using the light absorption images as shown in Figure 5A [59]. Such measurements are applicable for clinical evaluations, such as the detection of malaria through a point of care (LOC) device [60] as showed in Figure 5B. The system has also been used to differentiate healthy and infected RBCs using fluctuation or flicker spectroscopy [61]. The microfluidic fluctuation spectroscopy for cellular viscoelastic measurement is shown on Figure 5C [62]. On a similar note, dielectric spectroscopy can visualize and analyze RBC dynamics and flow in static conditions [63].

Another potential method for the analysis of blood cells is Raman spectroscopy. Since 1970s, it has become a popular technique for biochemical analysis of blood components such as RBC, WBC, platelets, plasma, serum, and the whole blood [64]. In parallel, it has also secured its place for multiple clinical applications such as stem cell research for cancer treatments [65,66], therapeutic drug analysis and infection detections [66,67], and the diagnosis of bacteria, cells, and tissues [68]. However, Raman spectroscopy suffers from a significant drawback due to scattering for ~100 dB weaker signals than those of fluorescence methods. In general, laser trapping along with the microfluidic platforms can help addressing this issue efficiently for detecting and analyzing specific biomolecules [69]. As a practical example, a combination of microfluidics, plasma fractionation, and localized Raman spectroscopy have been pursued. Furthermore, a set of observed spectral wavelengths can serve as a multi-parameter regression technique for analyzing plasma parameters from a single Raman spectrum [70].

Analysis of RBC and/or erythrocytes is a hot research topic by using microfluidic technologies for blood cell analysis. Studies on molecular guiding, sorting, and concentration of blood cells in microfluidic constructs have been proposed and experimented [71] with Raman markers to assess cell physiology and their responses to external stimuli and drugs [72]. For studies related to RBCs and platelets, key factors are sensitivity, reliability, and reproducibility of Surface-Enhanced Raman Spectroscopic (SERS) substrates. Additionally, different nanostructures such as nanotubes, nanorods, pyramid structures, and shell-isolated nanoparticles have been used to enhance the efficiency [73]. SERS spectra of normal blood samples have been analyzed with an active SERS microchannel for qualitative assessments utilizing the plasmonic enhancement of surface traps. Results indicate an increased intensity of Raman signals within the structured SERS area [74]. An optical guiding arrangement for Raman spectroscopy has been proposed in which RBCs are used for tailing the optical guide, and spectral analyses have been obtained from the excited resonant cells. This could be implemented for the demarcation of normal, met- or mixed RBCs [75]. Another study of Raman spectrum of a single RBC captured by a microfluidic chip was recorded to determine the conformation of hemoglobin under conditions similar to the hemodynamics of a blood vessel. Specific amplitude changes in the Raman spectrum reflect changes in pO_2_ due to O_2_ binding to hemoglobin heme. This result indicated that the Raman spectroscopy data obtained during the movement and arrest of erythrocytes can be used to assess not only the change in the hemoglobin heme conformation upon O_2_ binding, but also the redistribution of cells with oxidized and reduced hemoglobin, as well as to control the redistribution of oxygen concentration from cell to cell [76].

Furthermore, the infusion of sodium dithionite can result in enhanced oxygen intake capacities as well as the disorder of hemoglobin concentration within the RBCs as monitored via the UV-Visible and Raman confocal microscope [77]. Although Raman systems are highly sensitive and accurate, the methodologies are quite sophisticated at times and applications of multivariate techniques such as PCA (Principle Component Analysis) and LCA (Latent Class Analysis) could potentially increase the efficiency of the current systems [78].

Various attempts in microfluidic-based RBC analysis include novel techniques such as waveguide-mode sensors [79] for hemo-agglutination measurements as well as the restrictive channel method [79]. State-of-the-art optical tweezers have the potential for future applications of single-cell analysis. These advancements could be suited for RBC analyses in hemorheology, functional diagnostics, and therapeutics [80]. Finally, speckle based analyses have found various applications, including laser speckle de-correlation [81], erythrocyte aggregation [82], machine vision, and image processing [83], while contrast based imaging with specific observations can be used for the magnetic nanoparticle retention in the blood (Table 1).

## 3. Single RBCs in Microfluidics (State-of-the-Art in Industry and Academia)

### 3.1. RBC Dynamics of Fluid

The first and foremost consideration of microfluidic constructs is the analysis of fluid pressure with good accuracy. The main limitation is the required amount of fluid as input in the conventional pressure measurement instruments as the liquid amount in the microfluidic platform could be limited. In the area, researchers have proposed systems such as Servo Nulling Pressure Measurement System for microfluidics [85]. In terms of RBCs, the non-physiological stress on blood cells can cause inflammatory reactions, cell damages, and membrane ruptures. Simulation and analysis show that conventional calculations mainly use power-law-based models with poor accuracy. Simulations with calibrated models are much more susceptible to hematocrit alterations than those of conventional fluid dynamic analyses [86]. A common practice has used membrane properties with fitting experimental observations for theoretical and numerical predictions. In general, RBC simulations are classified into mesh-based, particle-based, or hybrid methods, while recent studies indicate that the local field-flow disturbances due to RBC can increase the dispersion of nanoparticles and platelets [87].

**Figure 6:**
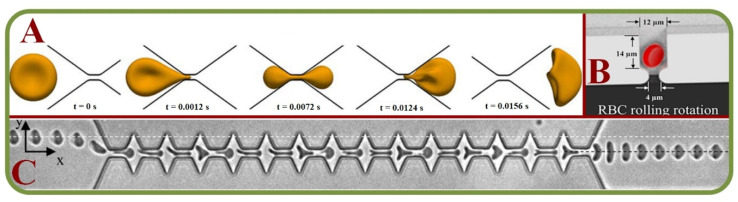
Single RBC trapping arrays. (**A**) Boundary simulations of a RBC squeezed through a submicron slit under prescribed inlet and outlet pressures [93]; (**B**) Independent and grouped single RBC rotations in a microwell for bioimaging applications [94]; (**C**) Microscopic images showing the shape recovery of single RBCs flowing out of a microfluidic constriction [92].

Studies on computational RBC models, single-cell mechanics, cells in large capillaries, RBC dynamics, and cell adhesions have all been actively pursued [88]. These include attempts to analyze RBC membrane compositions and architectures to allow the discriminations of the changes and effects [89]. Experiments on blood suspensions in low Reynolds number have demonstrated the Off-Centre Two Peak profiles (OCTP) of RBCs under microchannel flows [90] and RBCs can undergo multiple dynamics such as tumbling and rolling, followed by a combined rolling and tumbling effect to attain the poly-lobed shape [91]. RBCs flowing out of micro-channels mainly display two types of shape after the recovery process. Under the high viscosity and flow velocity, the impact of flow dominates the shape of RBCs. Under the low viscosity and flow velocity, the recovery time is reduced [92]. In one example, the RBC dynamics are analyzed through a submicron slit to obtain information on the cell deformation, transit time, and internal stress [93]. Figure 6A shows the boundary simulations of a RBC squeezed through a submicron slit under prescribed inlet and outlet pressures [93].

For adhesion and recovery measurements, a microfluidic device has been proposed to regulate flow through microchannels [95]. Pulsatile flows and continuous flows will affect the results, while the cell type responsible for this adhesion phenomenon varies with patients. It has been concluded that low-flows comparatively show more adhesive interactions [96]. In another work, a novel integrated system is used to assess the single-cell deformability index and detect the presence of distinct biophysical RBC subpopulations with high inter-patient variability of the Sickle cell disease [97]. Independent and grouped 3D single RBC rotations in a microwell for bioimaging applications have allowed novel studies by the hydrodynamic vortex flows as shown in Figure 6B [94]. Microscopic images of single RBCs flowing out of a microfluidic constriction is shown in Figure 6C [92]. Same techniques have been used to investigate the erythrocyte membrane interactions for the studies of cerebral capillary hyperemia [98].

### 3.2. RBC Agglutination/Aggregation in Microfluidic Environment

**Figure 7:**
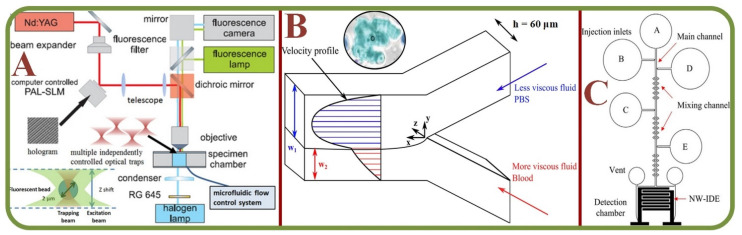
RBC Agglutination/Aggregation in microfluidic environment. (**A**) Laser tweezers combined with microfluidics for the single RBC dextran absorption study with the schematic layout of the holographic laser tweezer and diagram for fluorescence detection. [101]; (**B**) The RBC aggregation and the effect on non-Newtonian blood viscosity in a two-fluid low shear rate system. Inset: a sample of RBC aggregation with the “rouleaux” structure [105]; (**C**) The microfluidic chip structure of RBC agglutinations for the blood type detection [114].

Hemorheological properties are important aspects in the blood circulation and hemodynamic physiology. Microfluidic technologies have enabled the effective studies of RBC aggregation, blood viscosity, and other biophysical parameters. This is beneficial for assessing cardiovascular diseases such as stroke, coronary heart disease, and myocardial infarctions. Hemorheology is controlled by various properties, including the hematocrit composition, cell components, cell-free layers, and the plasma complex. RBC can aggregate close to the flow regimes such as tubes and walls, and there is a need to consider these factors to analyze how they influence the micro fluidic flows as discussed in this section. A newly proposed three-channel microfluidic platform has been used to analyze the aggregation based on the image intensity of aggregated and disaggregated channels [99]. To analyze the wall adhesion effects, circular microchannels have been employed to measure the wall to particle adhesion rates [100,101]. Figure 7A shows the combination of the laser tweezer and microfluidics for single RBC dextran absorption studies [101]. Results indicate a strong correlation between the RBC aggregation and the rate of adhesion. In a similar attempt to discriminate RBC aggregation and blood viscosity, syringe pumps have been employed with sequential image intensity measurements [102,103].

Analysis of aggregation under varying shear rates is yet to be fully studied, while the non-Newtonian behavior of blood under microcirculation have been useful in understanding the RBC aggregation [104]. Previously, studies have been made on the varying effects of temperature, hematocrit, shear rate, and viscosity of RBC aggregations [105]. Figure 7B shows that RBC aggregates under non-Newtonian blood viscosity at low hematocrit in a two-fluid low shear rate system, including the RBC shape of “rouleaux” (in the Inset) [105]. Similar studies have investigated the side effects and/or similar properties of erythrocyte aggregations, such as the influence of aggregation in mammalian species [106] and the analysis of surface-tension-driven blood flow with RBC aggregations [107]. Other relevant studies include periodic measurements of RBC aggregations, the rate of erythrocyte segmentation [108,109], the analysis of RBC aggregation under varying hematocrit concentration [110], the automation of microfluidic-based aggregation detections [111], the measurement of RBC aggregation in a continuum [112], and the optimization of microfluidic channels to improve the hydrodynamic dissociation of cell aggregations [113].

Agglutination of RBC occurs when there is a match between the antigen on the RBC surface and the blood serum antibodies. This is a standard testing procedure for detecting the blood type. Disposable microfluidic chips have been implemented using the principle of Electrochemical Impedance Spectroscopy (EIS) and electro-analytical measurements to measure the agglutination levels using Inter Digitized Arrays (IDA’s). The geometrical parameters, i.e., the number, width, length, and gap of the electrodes, can be optimized to improve the sensitivity of the IDE array, including by shrinking the electrode size to nanoscale [114]. Another real-time assessment method of blood agglutination uses passive bio-chips to induce a high agglutination rate for the effective match between the donor and the recipient [115]. Furthermore, the effect of RBCs aggregation in circular microchannels has been studied in terms of the aggregation index vs adhesion coefficient [100,101]. Figure 7C demonstrates the chip structure for the electroanalytical measurement of RBC agglutination for blood type measurements in a microfluidic system [114]. Another automated method for blood type determination by RBC agglutination assay through Tsoliclone monoclonal antibodies being introduce into the whole blood sample. The method proved that mechanical vibration of the sample promoted RBC agglutination and redistribution through the sample volume [116]. Semenov et al. assessed RBC aggregation and deformation by laser tweezers, diffuse light scattering, and laser diffractometry demonstrating the benefits of the optical methods for studying RBC aggregation deformability [117] while Trejo-Soto et al. studied microfluidics methods of assessing blood rheology in microcirculation [118].

Zhu et al. studying the OT (optical tweezers) trapped single RBC deformability exposed to low-level laser exposure (radiant fluence below 9.5 J/cm^2^) found that irradiated RBCs aggregate faster and the aggregates are easily destroyed by external influence, while high-level laser radiation (over 170.5 J/cm^2^ radiant fluence) triggers irreversible enhanced echinocyte formation. Also Zhu et al. claim that blood photobiomodulation mechanisms still remain unclear [119]. Another study established that suppression of RBC aggregation by osmotic swelling reduces the protein band formation in continuous Percoll density gradients. At the same time the aggregation suppression was found to cause a severe effect on compromising the RBC centrifuged age separation [120]. Another study of RBC aggregation stability asses the the distribution of RBC rouleaux shape and its adhesive nature towards endothelial cell in a non-flowing environment with biopolymer dextran present (known to be the RBC aggregation promoter). Authors observed constant rouleaux I-, L-, Y-shapes and clump-shaped with gradient change in dextran concentration (450–650 kDa) as well as dextran (7.5% and 10%) consistently promoting single RBC adhesion towards endothelial cell then aggregated RBCs [121]. Finally, the RBC aggregation has also been studied by using a microfluidic biosensor with continuously varying blood flows to determine the aggregation index vs shear rate dependency [112], and ultrasound-based methods for quantifying RBC aggregation [122].

Other studies covered RBCs aggregation and morphodynamics in patients with polycythemia vera and stroke [123] and COVID-19, which patients were reported to have RBC aggregation increased, along with blood viscosity despite lower hematocrit that heathy individuals while oxygen supplemented patients had even higher aggregation and blood viscosity than those without [124].

### 3.3. RBC Flow Analysis in Bifurcating Channels

In real blood microcirculation, the flow is characterized by a complicated network of micro-vessels that are branched systems rather than straight and straightforward tubes and RBCs are distributed or merged into different branches. The motion, aggregation, and deformation states have been widely studied with the following key parameters, RBC count, mechanical properties, and intracellular interaction strength [114].

**Figure 8:**
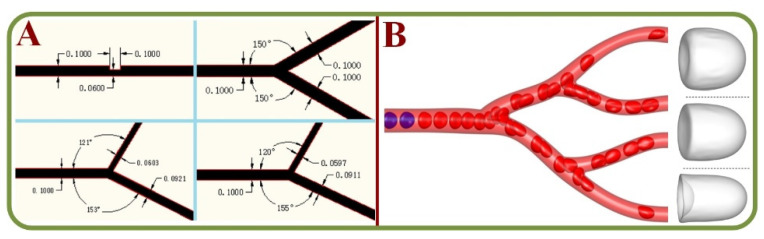
RBC flow analysis in bifurcating channels. (**A**) The analysis of pressure drop and flow behavior in hypertensive micro vessels with flow divisions in daughter branches of different bifurcated channels [127]. (**B**) Motion, deformation, and aggregation of multiple RBCs in three-dimensional micro vessel bifurcations with the diverging-converging bifurcations [128].

The aggregation of RBCs is the primary source for blood viscosity and it also affects the velocity profiles, local RBC concentrations, and cell-depleted layers at the channel walls. Therefore, studies have attempted to capture the cell-free layer variations in a microchannel network using high-speed cameras via automatic and manual methods [125]. To compare the variations of RBC and/or plasma protein concentrations, a T-shaped Microchannel has been loaded with blood samples and pushed by an air-compressed syringe for the continuous measurement of blood viscosity and erythrocyte sedimentation rates [126]. A study on the flow behavior and pressure within microchannels indicates a drift of RBCs from the center of the channel and the dependence of flow velocity on the inlet flow rate of the nitration with RBCs. Furthermore, a significant pressure drop has been observed due to increased viscosity [127,128]. Figure 8A shows the analysis of pressure drop and flow behavior in hypertensive micro vessels with flow divisions in daughter branches of different bifurcated channels [127]. Cell suspension models in complex micro-networks with inflow/outflow boundary conditions for RBCs in the microvascular network under different fluid velocity are shown in Figure 8B [128].

A finite element analysis for the thrombus formation in branched microcirculation has effectively predicted the areas of thrombus nucleation for the potentials of fluid stagnation, bifurcations, and recirculation based on velocity, shear rates, and cell distribution profiles [129]. Studies have also assessed the migration properties of deformed cells using a Y-shaped micro channel with bifurcation angles between 30 to 180 degrees. The trapping of blood cells within the Y-channel have been found to be highly efficient at 120 degrees, and the trapping efficiency for white blood cells has increased in comparison to that at an angle of 60 degrees [130]. Margination and cell phase separation have been independently assessed for their efficiency in rectangular and irregular channels instead of circular channels [131], as well as the effects of capillary dilation [132]. Microfluidic-based biosensor for the blood viscosity and erythrocyte sedimentation rate analysis have also been studied previously [126].

### 3.4. RBC Dielectrophoretic Analysis

In scientific terms, dielectrophoresis refers to the movement of dielectric particles when subjected to a non-uniform electric field. This phenomenon is widely used in the manipulation, transportation, sorting, and separation of particles. Biological cells also possess dielectric properties. Initial studies in this regard have focused on microfluidic platforms for cell trapping and rotation-based analyses, including rolling-based rotation of single cells and electrokinetic approaches for the rotation of cell clusters [94]. The Dissipative Particle Dynamics (DPD) method has been used for the simulation of particle trajectories in microchannels, including the prediction of the RBC trajectories in the presence of dielectrophoretic force [133]. In general, RBCs have been found to move towards high electric field gradients and undergo morphological deformation in certain conditions under the influence of dielectrophoretic forces [134].

**Figure 9:**
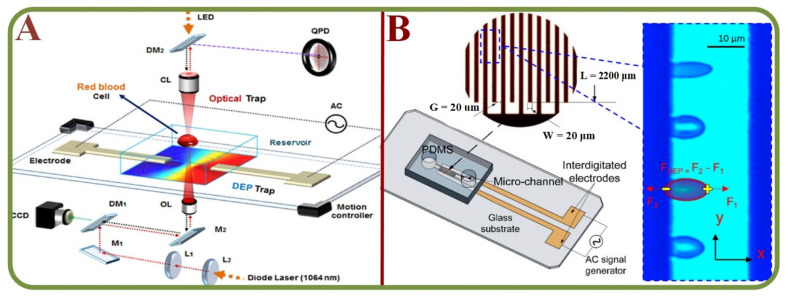
RBC Dielectrophoretic Analysis. (**A**) The system schematics of dielectrophoretic force measurement of RBCs exposed to oxidative stress using optical tweezers [140]. (**B**) The dielectrophoresis testing of nonlinear viscoelastic behaviors of human RBCs, including the biomechanics testing schematics of live cells using the dielectrophoresis effects [143].

Technologies based on the dielectrophoresis effects are effective for the analysis of fatigue, force, and stress at the cellular level. For example, cell manipulation systems have been widely employed to measure cellular biomechanics using microfluidic platforms, including studies on cell stretching and manipulation [135,136], electrical property changes of stored RBC [137], label-free and noninvasive characterization for the viscoelastic properties of RBC [138], benchmarking dielectrophoretic separation metrics of unknown types of RBC (healthy, modified, …) [139], the oxidative stress analysis for RBCs (Figure 9A) [140], dynamic fatigue measurements [141], detecting circadian rhythms in RBCs [142], nonlinear viscoelastic analyses (Figure 9B) [143], biomechanics of erythrocyte membrane failures [144], liquid metal electrode-based dielectrophoretic schemes [145], and a portable system with multiple dielectrophoretic applications for RBC analyses [146].

### 3.5. Deformation of Single Erythrocytes in Microchannels

**Figure 10:**
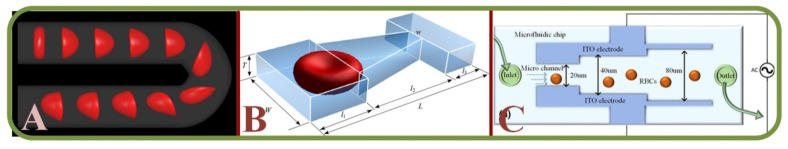
Deformation of single erythrocytes in microchannels. (**A**) Single RBC deformation sequences in a curved micro-vessel [152]; (**B**) A microfluidic chip for studying the critical pressure to drive a RBC through a contracting microfluidic channel [160]; (**C**) The microfluidic system to measure the RBC deformability with parallel electrodes in a microfluidic manipulation system [159].

The RBC deformability is a unique and common feature that allow cells to travel through small capillaries. This happens due to the combined functional effects of structural alternation of cytoskeletal proteins, intracellular ions, water, and the membrane-surface to volume ratios [147]. Observing such changes could help the identification and analysis of disease pathophysiology as well as RBC disorders. The analysis of RBC deformability in micro capillaries is a novel way of assessing confined flow behavior such as viscoelastic properties [148], shear moduli [149], and changes in bending stiffness [150]. Other conventional methods of assessing cell deformability have utilized individual cell analyses (i.e., pipette and optical tweezer) or bulk analyses (i.e., ektacytometry and multiple channels) [151]. Studies using the particle dynamics simulation have shown that small curvature does not affect RBC flow but an increase in the diameter of curvature induces a secondary flow, where the axial velocity is skewed towards the inner wall of the micro vessel [152]. Figure 10A shows the motion and deformation of a single RBC in a curved micro-vessel with its shape evolutions (arch, ripple, spiral, U-bend) [152]. Novel devices have been used to efficiently detect changes in the deformability ratios of RBCs to study the kidney disease [153]. Furthermore, multiple devices are available to quantify the biophysical parameters of RBCs, including the deformation, aggregation, and hematocrit concentrations [154].

A key challenge in the domain of transfusion medicine is the development of an efficient method for the discrimination of low-quality units to enable the possibility of real-time assessment. Researchers have proposed a multiplexed fluidic system that can measure the deformability index based on the pressure required for the microcapillary transition [155]. In order to quantify the differences in the length of packed RBCs, researchers have devised a microfluidic construct to evaluate RBC deformations in centrifugal motions. The term ‘compressibility index’ (CI) refers to the deformation levels of normal and hardened RBCs [156]. Significant differences in the distribution of flow velocities and hematocrit concentrations have been noted within deformable and rigid RBCs in straight microchannels [157] due to the complex cell-cell interactions. Analyzing the changes in the shape of RBC under varied flows and channel dimensions can enable the characterization of RBC under shear elasticity for the qualitative assessment of the mechanical properties in RBCs.

According to the protocols set by FDA, RBCs can be stored for a maximum of 42 days under refrigerated conditions. While some RBCs may initiate early degradation, they tend to deform to induce microcapillary obstructions and post-transfusion RBC clearances. A deformability cytometer has been employed to study these characteristics to understand the blood storage injury, along with an arrangement to remove less deformable RBC subgroups [158]. An approach using electrode-based microfluidic systems has studied RBC stretching using dielectrophoretic forces. This also elucidates the relationship between the dielectrophoretic forces, voltage, and electrode gap distances at the single-cell level [159]. A pressure gradient-based approach is also beneficial in the assessment of cell rigidity. A numerical simulation method has been implemented to assess the relationship between pressure requirements to push RBCs through microchannels [160]. High-throughput microfluidic characterization devices for the erythrocyte shapes and mechanical variability have also been studied [161]. For example, a novel “plunger device” has been used to study the microfluidic deformability of RBC storage lesion [155]. Figure 10B shows the critical pressure for driving a RBC through a contracting microfluidic channel [160]. and Figure 10C shows the system structure of RBC deformability measurements using parallel ITO electrodes in a microfluidic manipulation system [159].

Among hyperbolic, smooth, and sudden-contraction-based microchannels, the hyperbolic channels show enhanced extensional flow with a homogeneous strain rate along the centerline [162]. Similar to previous studies, an attempt has been made to measure both the viscosity and elastic constant using a feedback-based cell manipulation technique together with the computational analysis [163]. These efforts have yielded the measurement of Young’s modulus by estimating the absolute values of the viscous and elastic constants [164]. The shear modulus distribution has also been simulated to construct deformability-based figure of merit, followed by image processing algorithms to identify and track RBCs’ position and shape [165]. Furthermore, the testing of micro beads has been used to enhance the repeatability and precision of a microvascular analyzer to assess the RBCs deformability [166]. To demonstrate the viability of the RBC subpopulation, a cell-to-liquid interface has been implemented along with a disposable air-compressed pump as the primary source for the blood supply. However, due to clogging, fluctuations occur at the cell-liquid interface pressure channel [167].

Recently, it has been found that mesoscopic hydrodynamic simulations can describe the nature of cell behavior in a complex microfluidic flow to assess RBC biomechanics [168]. For example, a dynamic deformability indexed RBC sorting algorithm via image processing can help comprehend different vaso-occlusion events for dynamic studies [169]. In another example, a complex microfluidic-based plunger system has been found to provide controlled pressure precisely to squeeze RBC in the assessment of deformability [170]. Similarly, an air-cavity-based syringe system has been used to study the variations in blood flow velocities and the image intensities of clogged RBCs [171]. Mathematical models of deformable RBC with Discrete Element Methods have also been taken into account, along with the lattice Boltzmann models of immersed boundaries [172,173]. Other relevant studies include the assessment of shear dependencies in RBC adhesion [174], analysis of the changes in RBC stiffness [175], mechanical characterization of stored RBCs via mathematical models [176], donor-dependent aging curves based on microfluidic RBC models [177], effects of channel geometry in RBC sorting [178], and the assessment of RBC deformity using iron-dextran tests [179].

### 3.6. Miscellaneous Observations

Similar to aforementioned studies, the analysis of hematocrit in microfluidic platforms is becoming a common trend. Experiments related to the heterogeneity in RBC distribution indicate a non-uniform nature in microfluidic platforms. These are characterized by local hematocrit gradients and are assumed to impact the cellular oxygen distribution [180], as well as the relationships between RBC deformability and hematocrit partitioning in bifurcating channels [181].

The application of paper-based microfluidic devices favors the studies on the feasibility of RBC and hematocrit measurements and holds great potential for remote regions with limited resources [182]. Miscellaneous applications of microchannel flows include the analysis of high-speed flows [183], RBC dynamics under oscillatory flows [184], structural and functional assessment of erythrocyte membranes [185], analysis of RBC water permeability [186], response to loading and stress [187], factors influencing RBC homeostasis and pharmacological interventions [188], and applications of vascular microfluidics to blood-endothelium interfaces [189]. Other studies include membrane-based microfluidics for separation [190], effects of osmolality and perfusion on erythrocyte rheology [191], enhanced deposition analysis of sickle cell RBCs [192], and studies on RBC capillary velocities as a function of oxygen content [98]. Electro mechanic experiments include observing rabbit RBCs in optofluidic tweezers and stretchers [193], impedimetric ratio measurements via microfluidic chips and ZnO nanowires [194], blood platelet enrichment methods via surface acoustic wave (SAW) microchannel platforms [195], and MOSFET-based microfluidic gates [196].

## 4. Clinical Implications of Microfluidic Based Single RBC Analysis

Malaria, cancer, sickle cell diseases can cause a wide range of unwanted physiological and biomechanical alterations often at the cellular level. Current state-of-the-art diagnostics schemes heavily rely on molecular, immunological, and pathological assessments. The pathophysiology of human diseases can be classified based upon intrinsic properties and pathophysiological changes, either morphological, biomechanical, phenotyping, cell enrichment, or separation-based [197]. Accurate diagnosis is possible only when the ailment is recognized correctly. Point-of-Care Testing (POCT) can test patient specimens with faster and remote diagnostic options. It has been shown to effectively detect proteins, nucleic acids, cells, metabolites, and communicable diseases such as HIV, Strep A/B, malaria, and meningitis [198,199]. With extensive growth and advancement, microfluidic technology is proving to be a competitive methodology in terms of accuracy and efficiency. These microfluidic systems are mainly categorized into three types, i.e., Optical-detection based (fluorescence, colorimetric, chemiluminescence, lens-less shadow imaging), electrical signal-based, and centrifugation-based devices [200]. Recent advances in these domains further include thread-based [201], compact disc or DVD-based [201,202], and paper-based [201,203] constructs. POCT applications for infectious diseases are also progressing rapidly, while the major challenges are in producing efficient, inexpensive, and self-reliant technology. The current initiative focuses on advancing POC-based cell counting, antigen/antibody tests such as lateral flow assays (LFAs), agglutination assays, paper-based microfluidics, 2D paper networks, and nucleic acid base biomarkers [204].

**Figure 11:**
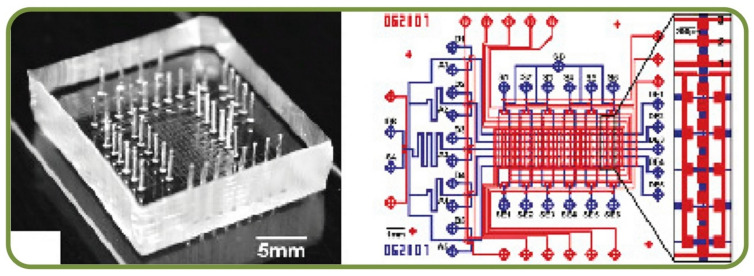
The internally calibrated quantification of protein analytes by fluorescence immunoassays in a disposable elastomeric microfluidic chip [206].

Some other clinical advances of microfluidic based single RBCs are microfluidic device for studying the deformability change of stored RBCs (centering, orienting, folding, and shape recovery) [205], internally calibrated quantification of protein analytes in human serum using fluorescence immunoassays in a disposable elastomeric microfluidic chip [206] shown in Figure 11, a microfluidic dielectric sensor for the Point-of-Care assessment of hemostasis [207], and a holographic optical tweezer setup for measuring the RBC interaction force [208].

Another recent work has demonstrated the modeling of biomechanics and biorheology of RBCs in Type 2 Diabetes Mellitus and the stretching response of different RBCs [209]. The novel “cross-bridge”-induced interaction of RBCs has enabled the studies by optical trapping and the process of attaching 2 RBCs [208]. Fast ferritin immunoassay has also been studied in a PDMS microchip [210]. Another example is the multi cargo-loaded RBC micromotor and magnetically guided and ultrasonically powered transport behaviors in a microchannel [211]. Furthermore, a successful example of micro-physiological model of RBC has enabled the measurement in fresh RBC units from the control and transfusion groups in the presence and absence of cyclic stretch [212].

Blood microcirculation behaviors have been analyzed from the perspectives of a water-inside-a-glass-tube model and studies have shown a direct dependence of RBC dynamics on the shear force across blood vessels. For example, analysis of the malaria pathophysiology has indicated an increased adhesivity and decreased deformability in infected cells. In the sickle cell disease, severe shape deformation in RBCs occurs due to a lack of oxygen. These facts been applied in microfluidic devices by analyzing cell density, hypoxia, and the effects of vaso-occlusion and adhesion of RBC cells [213]. The whole blood fractionation has been shown as a versatile method to separate the blood in POC devices but this comes with the challenges in flow dynamics as RBCs can be ruptured due to shear stress and lead to hemolysis [214]. On the other front, lateral flow strips (LFS) assays have been widely employed for various studies, such as biomolecule pathogenesis, cancer cells, bacteria, and viruses. Disease diagnostics can be accomplished through the analysis of cell behavior, heterogeneity, migration, angiogenesis, cell-cell communications, molecular profiling, single-cell epigenomics, transcriptomics, and proteomics [203,215]. Advanced microfluidic technologies in diagnostics also include assessing microcirculatory parameters on bio-impedance-based microfluidic devices [216] and studies on the assessment of malaria, sepsis, HIV, SARS, dengue, and tuberculosis [217].

### 4.1. Analysis of RBCs Sedimentation Using Microfluidics

**Figure 12:**
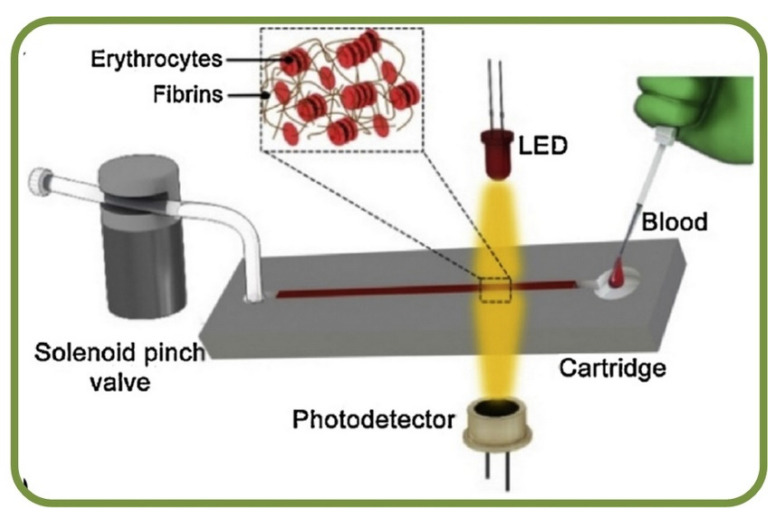
Optofluidic point-of-care device for the quantitative investigation of erythrocyte aggregation during coagulation [221].

Erythrocyte sedimentation rates (ESR) are clinical tests to analyze erythrocyte deposition rates for inflammatory diseases such as temporal arteritis, polymyalgia rheumatica, stroke, heart attack, and prostate cancer. Studies has indicated that the erythrocyte aggregation rate is an efficient marker for the prediction of ESR, and it leverages the relation between particle sizes and sedimentation rates. Microfluidic systems can measure the steps of disaggregating erythrocytes followed by re-aggregation process, which is monitored using a NIR light source. The ESR is calculated based on the changes in the optical signals and results also show a very high correlation coefficient of about R^2^ = 0.86 [218]. Furthermore, a vertical microfluidic system embedded with a cell-tracking system has been implemented for imaging to provide an edge over conventional analysis by reducing the sample volume and the analysis time [219]. RBCs also can form face-to-face branched or linear structures under static conditions or low-shear rates in the presence of appropriate macromolecules and are termed as ‘rouleaux.’ This process is reversible, and the cells can disintegrate by applying shear forces. The most common RBC aggregated disorders are hypertension and thrombosis. Microfluidic measurements of erythrocyte sedimentation rates have been studied using the finite element method [220]. Furthermore, optofluidic assessments have provided great insight into the erythrocyte aggregation and coagulation, thereby estimating the time in the formulation of fibrin networks as a marker equivalent to the time of coagulation [221]. The assessment of fibrinogen molecule interaction with RBCs has utilized techniques, including laser aggregometry, flow cytometry, and optical tweezers within microfluidic platforms [222]. Figure 12 shows an optofluidic point-of-care device for quantitative investigation of erythrocyte aggregation during coagulation with the schematics [221]. Another example for determining the erythrocyte sedimentation rate has enabled research of settling RBCs in terms of the diameter of cells and the population of cell/rouleau of different sizes [219]. Furthermore, the idea of combining the benefits of laser aggregometry, flow cytometry, and optical tweezers has been successfully implemented for the assessment of fibrinogen macromolecules interaction with RBC membranes [222].

### 4.2. Analysis of Malaria using Erythrocyte Based Microfluidics

At present, more than 3 billion people are at the risk of contracting malarial infection, a parasitic disease that accounts for a yearly death toll of 1 to 2 million. Malaria-infected RBCs can be characterized by the different stages of the ring, trophozoite, and schizont shapes. The progression of this infection induces biochemical, optical, and morphological changes of infected RBCs, making them thicker, rigid, and increasingly viscous. Furthermore, the deformation index as a suitable biomarker for assessing malaria-infected cells in microfluidic platforms was studied [219] as well as the system for dielectric characterization of Plasmodium falciparum-infected RBCs with microfluidic impedance cytometry [223].

While the most crucial challenge in medical diagnostics is the development and deployment of high-performance assays for low resource settings, paper microfluidics provides high sensitivity and lower Limits of Detection (LOD) as compared to those in the conventional Lateral Flow Tests (LFT) [224]. For example, currently available devices for detecting malaria includes the Rapid Diagnostic Tests using microfluidic platforms for reading Plasmodium parasites with multiplexed DNA-based malaria detection methods [225]. Further reports have devised microchip systems with a push column for RBC recovery and a fluorescence detector for malaria parasites. Correlations with optical microscopic observations and linear regression-based analysis indicate an R^2^ value of about 0.9945 [226]. Another study of malaria parasite proteins on the surface of infected RBCs and their potential anti-malaria adhesion-inhibiting molecules was conducted with the high-throughput screening approach rrevealing promising leads for anti-adhesion drugs synthesis [227].

On the other hand, microfluidic cell-phoresis has demonstrated the high throughput measurement of RBC deformability for malaria screening for drug discovery applications [228] as well as chips for studying plasmodium falciparum merozoites interaction with the RBC membrane during invasion to modulate RBC deformability and facilitate invasion [229] Lastly, impedance-based analysis have been shown to efficiently demarcate uninfected and infected RBCs based on cell membrane properties as good prospects for the label-free sorting process [223].

### 4.3. Analysis of Sickle Cells Disease Using RBC Microfluidics

**Figure 13:**
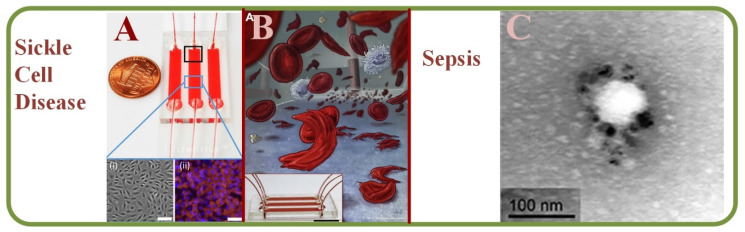
(**A**) Endothelium-on-a-chip for probing the RBC adhesion to heme-activated endothelial cells to reflect clinical phenotype in sickle cell disease, including blood sample images [232]. (**B**) A SCD biochip for RBC adhesion assay monitoring, including the SCD RBCs suspension [230]. (**C**) An image showing the plasma-derived extracellular vesicles on the RBCs deformability in polymicrobial sepsis [234].

Sickle cell disease (SCD) has been characterized by the abnormal cell adhesion to the endothelium and microfluidic evaluation of RBC adhesion can help identifying membrane damages as well as aberrant activations at multiple receptor sites [230]. Additionally, microfluidics also helps in studies related to SCD therapeutics [231]. Specifically, endothelialized microfluidic platforms can monitor cell adhesion and simulate intravascular SCD. While the adhesion rates may vary within subjects, RBCs mainly adhere to those that tend to haemolyse with complex heterocellular adhesive interactions [232]. Figure 13A shows the endothelium-on-a-chip for probing RBCs adhesion to heme-activated endothelial cells to reflect clinical phenotype in SCD, including blood samples images [232]. Furthermore, studies can quantify the viscosities of whole blood samples and results have found higher viscosity in SCD than those in normal hemoglobin samples [233]. Figure 13B shows a sickle cell biochip photo and the picture of the SCD RBCs suspension [230]. The photo of the effect of plasma-derived extracellular vesicles on the RBCs deformability in polymicrobial sepsis is shown in Figure 13C [234].

### 4.4. Sepsis Diagnosis Using RBC Microfluidics

Clinically, sepsis is the inability of the human body to release chemicals that fight against the invading pathogens. RBC abnormalities are significant but early markers that can express the abnormal alterations in RBCs due to sepsis is yet to be discovered. Experiments such as the lippo-saccharide induced sepsis model have been used to investigate RBC abnormalities for early detection of sepsis injuries, including parameters such as aggregation indices, aggregation half-time, and elongation indices [235]. Spontaneous motility assessment of neutrophils has also been a suggested marker for sepsis in the presence of blood plasma, while a machine learning-based scoring system has been shown to identify sepsis patients [236]. With the help of laser diffraction and microfluidics, RBC deformability has been analyzed in a poly-microbial sepsis model, along with an attempt to identify the causes [234].

### 4.5. Cancer Diagnosis with RBC Microfluidic Systems

**Figure 14:**
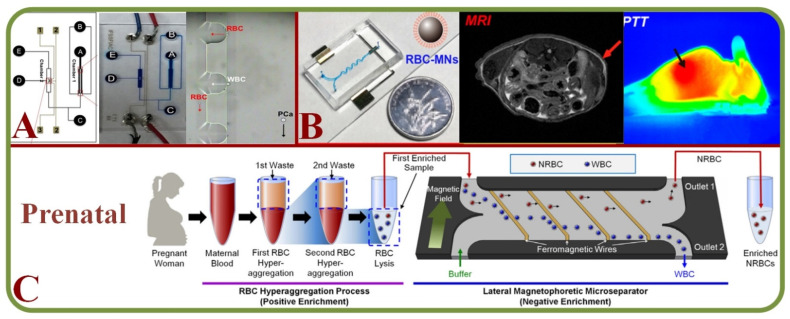
(**A**) Structure and schematic of the microfluidic chip for the isolating prostate cancer CTCs from RBCs and WBCs and single-cell measurement of drug accumulations [239]. (**B**) A microfluidic device for guided cancer therapy with the synthesis of RBCs with membrane-coated magnetic nanoparticles, including the fabricated chip, in vivo tumor MRI with RBC magnetic nanoparticles, and in vivo IR thermal images of a tumor bearing mice [242]. (**C**) A 2-step cascade enrichment procedure for the isolation of nucleated RBCs using enrichment processes based on the RBC hyper-aggregation and lateral magnetophoretic micro-separator [243].

Tumor cells are a mix of complex and heterogeneous entities and the microenvironments are generally composed of different cell types, including fibroblasts, adipocytes, endothelial and immune cells. Droplet-based microfluidics such inDrop and Drop-Seq have been employed for single-cell RNA sequencing as microfluidic systems can capture the size of a wide range of cells effectively [237]. In addition, markers and label-free cancer cell detections have been the focus in recent years. For example, an acoustic cell separation platform has been proposed for the enrichment and isolation of cancer cell recovery and high purity analytical applications [238]. Along with the analysis and visualization of tumor cells, studies related to drug accumulation and response have been pursued with the isolation of prostate cancer CTCs from RBCs and WBCs as shown in Figure 14A [239] and to fluorescently tagging CTCs for magnetic isolation [240]. Key works in this area include RBC mimicking micro-platforms (micromotors) for photodynamic cancer therapy [241] and the synthesis of erythrocyte-coated magnetic nanoparticles for image-guided cancer therapy in Figure 14B [242], where a microfluidic device is shown for cancer therapy with the electroporation-facilitated synthesis of RBCs and membrane-coated magnetic nanoparticles. This figure also includes the fabricated chip, in vivo tumor MRI with RBC magnetic nanoparticles, and in vivo IR thermal images of a tumor bearing mice.

### 4.6. Prenatal RBC Diagnosis

Prenatal diagnosis has valuable information on the fetal health and efficient methods to separate fetal cells from the maternal circulation are important. Microfluidic technologies have enabled efficient cell separation with additional benefits of small samples, low cost, versatile design, and automation [244]. A novel silicon-based micro-platform has been implemented for capturing fetal nucleated RBCs for cell-based non-invasive diagnosis [245]. A two-step cascaded enrichment methodology has also been proposed to isolate nucleated RBC in maternal blood using the principle of RBC hyper-aggregation and negative enrichment in microfluidics [243] (Figure 14C).

### 4.7. Miscellaneous Areas for RBC Clinical Implications

RBC-based microfluidics chips have shown prospects in areas of the clinical treatments and diagnostics. Common RBC disorders such as sickle cell disease, hereditary spherocytosis, and diabetes have been characterized by alterations in shape and/or size due to protein mutations, or changes in the extracellular environment, leading to abnormal cell deformations, impaired cell stability, and increased aggregation. Various studies include experimental (microchip fabrication, flow geometry design, and measurement) and computational methods (Finite Element, Finite Volume, Immersed Boundary, Arbitrary Lagrangian-Eulerian, Dissipative Particle Dynamics, Boundary Element methods) [246]. Computational simulations have modeled diabetic complications such as diabetes mellitus [209] and diabetic retinopathy [247]. Direct methods include the hemoglobin detection [248], transfusion-induced pulmonary vascular injury [212], and serological applications like targeted exosome sequencing and profiling [249,250]. New studies include the effect of drugs (Poloxamer 188) on RBC membrane [251], assessment of temporal variations in rheological and platelet adhesion [252], diagnosis of anemia [253], optical trapping of cross-bridged RBC interactions [208], isolating intact bacteria from the blood via selective cell lysing [254], theranostics [211], and dielectric sensors for point of care analysis [207]. Finally, microfluidic devices for RBC assessments in clinical practice are summarized in Table 2.

## 5. Microfluidics Based Red Blood Cell Sorting

Conventional blood sorting is made by analyzing the physical properties between the different blood cells or biological properties. These methods are highly efficient but there are notable shortcomings such as prolonged processing time, cost factors, limitations in suitable antibodies, and the amount of blood required for the protocol [257].

**Figure 15:**
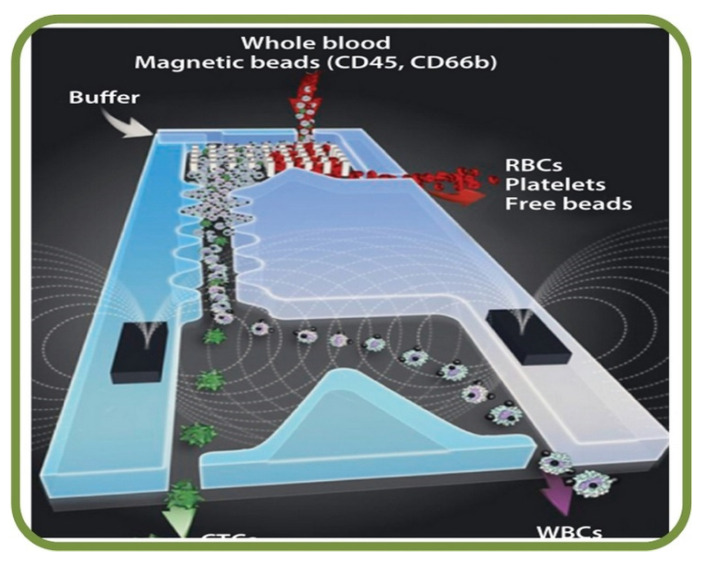
Microfluidic blood sorting devices. Biomimetic microfluidic chip for separating malaria-infected RBCs from healthy RBCs [264].

Among the recent examples from literature of microfluidic RBC sorting devices are the enhanced separation of aged RBCs in a microfluidic device and cells marginations [258], RBCs filtration techniques using slits, pillars, and weir barrier [259], the separation of crossflow RBCs form WBCs isopore microfilter [260], the microfluidic separation of RBCs and CTCs (labeled with magnetic particles functionalized with EpCAM for the immunomagnetic detection) [261], the high-throughput and cogging-free microfiltration platform for the separation of whole blood RBCs, WBCs, etc. [262], and the single RBC acoustic separation using standing surface acoustic waves (SAWs) [263]. Finally, Figure 15 shows the biomemetic microfluidic chip for separating malaria-infected RBCs from healthy RBCs [264].

The field of blood sorting using microfluidics has been evolving by scaling down the operational volume and optimizing the device parameters. Table 3 summarizes different microfluidic-based sorting techniques in two categories: precise sorting or bulk/large volume sorting. Precise sorting incorporates methodologies like acoustophoresis, dielectrophoresis, optical methods, and MEMS-based approaches (piezo actuators, vapor bubble actuators). Large amount scale sorting involves active methods (acoustophoresis, dielectrophoresis, magnetophoresis), and passive methods (micro-filtration, inertial separation, and deterministic lateral displacement). In recent years, hybrid systems that can incorporate both types are being demonstrated [265]. The sorting processes employ methods such as microfiltration, hydrodynamic-based sorting, affinity-based (e.g., magnetospheres based), acoustophoretic, biomimetic separation, and integrative systems [266]. Various clinical implications have been considered, including methods for rare cell enrichment, intraoperative blood salvaging, extracorporeal blood purification for sepsis therapy, wearable or implantable artificial kidneys, cleansing of banked blood for allogeneic transfusion, and wide-scale cell transfusion techniques. In general, separation techniques are either passive or active and the passive methods [267] do not rely on external force and are comparably less complex. Additionally, label-free techniques are useful when dealing with heterogeneous cell populations and offer high sorting specificity [268]. So far, research attempts have separated various blood components such as RBC’s [267], neutrophils [269], and blood plasma [270].

### 5.1. Cross Flow Filtration of Single RBCs in Microchannels

During the microfiltration process, the fluid flows on top of the membrane surface to allow the permeation due to a pressure difference. Therefore, an efficient filter requires well-defined cross-sectional area, axial flow rate, and a specified fluid that can produce the required axial pressure gradient [272]. Studies have focused on simulating and regulating the trans-membrane pressure to enhance the filtration capabilities. This is further proceeded with experiments to characterize the impact of filtration rates, transmembrane pressure, and shear rates to the dynamics of erythrocytes during the filtration process. It is found that erythrocytes tend to roll under low shear while settling under high filtration rates [273]. In one example, a microfluidic filtration platform with integrated rotary and bidirectional micro-pump has been used to separate WBCs from the whole blood with high efficiencies [262]. Similarly, fetal RBCs have been separated by a microsphere-assisted microfluidic device with high purity and viability [274]. Other relevant studies include human monocytes with size-selective trapping in microfluidics [275] and the spectroscopic assessment of cross-flow filtrations [276].

### 5.2. Blood Cell Counting and Sorting with Microfluidics

Disposable and low-cost microfluidic platforms for the automated blood cell counts are desirable in rural and remote regions. A comparison of cell counting efficiencies between an automated microfluidic platform, a hemocytometer, and conventional techniques such as the golden standard of flow cytometry have indicated a good correlation [277]. Another work involves the integration of a microfluidic cytometry with on-chip optical systems to detect cells in biological samples [278]. This field of studies have focused on the cell separation and deformability using cross-flow microchannel networks [279]. The next generation microfluidic devices could add additional conveniences such as: improved sorting and accuracy, capability to process native biological fluids and diverse types of cells, multiplexed sorting with reduced aerosol and biohazard risk, and compactness for mobility and operational ease.

The current state of the art cell sorting techniques focus mainly on three types: fluorescent label-based, label-free, and bead-based schemes [280]. Dielectrophoresis is an efficient method to separate single cells, specifically RBCs and/or erythrocytes with high sensitivity with the capability of label-free analysis based on the dielectric properties of target molecules. A proportional relationship has been found between the electrode pitch and strength of the electric field, while the dielectrophoresis is proportional to the cubic radius of the particles (i.e., cells) [281]. In a separate but similar experiment, human RBCs have been sorted from polystyrene beads using the dielectrophoretic separation method [282].

A self-filling device has been proposed to automatically separate unprocessed human blood, along with Raman analysis for non-invasive manipulation of RBCs [283]. Viscosity-dependent margination techniques are widely utilized in cell separation experiments. The margination characteristics of deformable RBCs are somewhat influenced by the cytoplasmic viscosity. Results indicate the tendency of RBCs to traverse into areas of stable equilibrium in the absence of cell-cell collision [284]. However, the conventional microfluidic platforms are prone to have mismatch volume while being interfaced with macro-scale analytical instruments. Inertial microfluidic devices alleviate these problems by providing label-free and high throughput separation for POC and bedside assessment applications [285]. The density-gradient-based microfluidic platforms provide label-free assessments and minimize the activation of isolated cells as compared to other methods [286]. Similarly, viscoelasticity-induced lateral migration techniques have been employed in blood component margination for non-Newtonian fluids, along with the analysis of the flow rate efficiency on leukocytes within the erythrocyte bulk [263].

Centrifugal systems are not limited to blood component analysis but are also applicable for immune cell analysis from the blood within laboratory settings with a centrifugal microfluidic chip [287]. Stiffness-based characterization of single cells is also a viable method widely implemented in cell sorting applications, including stiffness-based demarcation [288] and Deterministic Lateral Displacement (DLD) type devices [289] in the deformability-based sorting. The DLD technique has proven itself as an efficient method as many experiments have been pursued based on its principle of the hydrodynamic force, in which the larger particles tend to be in the lateral direction while the smaller particles continue within the flow regime. A mathematical expression for flow analysis has been derived using an integral equation solver for the vesicle flows [290]. The dynamical properties have also been considered to relate the effects of device geometries and viscosity in the intracellular fluids and the suspending medium [271]. Another study demonstrated the hydrodynamic separation method of parasitic T. Cyclops from human RBCs where the effective dimensions of the parasite depend on its orientation in the flow [291]. Within concentrated erythrocyte suspensions, particles can encounter numerous types of internal collisions and are highly dependent upon the ratio of particle to device dimensions [292].

The non-proportional distribution of RBCs can cause complications with the microfluidic channel bifurcations and this requires further study [293]. One approach is a micro-milling process for the production of microchannels with dimensions lesser than 30 µm for the efficient separation of RBC from plasma membrane [294]. Another enhanced separation techniques utilize the channel cross-section areas for the separation of stiffened cells with improved efficiency [258]. Furthermore, RBCs and WBCs have been separated using a hydraulic-based microparticle technique [295]. With the controlled flow design, trapping and release of microparticles are distinctly visible. Following the bifurcation, cross-flow, and hydrodynamic principles, a microfluidic device has been implemented for processing the whole blood processing to extract the plasma, WBC, and RBC [296]. Future prospects in this area include rapid isolation of blood plasma for quantification of proteins [297], image-based sorting [298], extraction and classification of morphological elements from human blood using optical coherence tomography [299], label-free analysis via AC-impedance and light-scattering flow cytometry [300], non-destructive identification and isolation using optical tweezers [301], and magnetic force-based separation of infected cells [302].

## 6. Microarrays and Single RBC Trapping Techniques

Platforms based on microfluidic array can employ static culturing of adherent cells for the dynamic control of fluid perfusion while facilitating hydrodynamic trapping of cells [303]. PolyDiMethylSiloxane (PDMS) has been the primary choice as the material. Various geometries for efficient cell capturing have been tested, including U-shaped constructs, flow shortcut structures, micro-cavity-based traps [304], patch-clamp-based array chips [305], microwell arrays or microarrays [306], and microfluidic-based hydrodynamic trapping [307,308,309]. Other single-cell trapping methodologies include dielectrophoretic-, chemical-, gel-, magnetic-, acoustic- and optical-trapping schemes [308].

### 6.1. The Concept of Microarrays

Microarrays are microfluidic platforms targeting applications of single-cell analyses and manipulations. They are popular due to their advantages in scalability, cell capture ability, and compatibility for imaging applications. In general, the cell patterning technology has been widely used to analyze and understand fundamental cellular properties such as cell migration, polarization, differentiation, proliferation, and cell signaling. It often combines with other advanced schemes for applications in tissue engineering, neuronal-network, cell-based biosensing, and drug screening. The most common cell patterning methods include inkjet printing, optical-tweezer, dielectrophoresis, and laser-guided direct writing to place cells at specific locations via external forces. Less common methods include capturing and confining cells using microchannels, micro traps, and the selective attachment of randomly seeded cells onto adhesive materials, which is also called chemical patterning. Some of these platforms are advantageous as they eliminate the need for cell repellent materials [303,310]. For example, a combination of hydrodynamic and dielectrophoretic microfluidic systems has been used to separate blood plasma from fresh blood using a microchannels for RBC trappings [311]. Similar platforms integrated the electro-active micro-well array with barriers capable of capturing and holding single RBC [312], and arrays of sequential hydrodynamic single cell trapping structures [309]. Furthermore, the IR laser capabilites to form a bubble to displace trapped cell/particle

Deformability analysis of RBCs using micro-capillaries has shown experimental observations based on the RBC dynamics to detect cellular subpopulations [171]. Other prospective studies include, RBC shape assessment [313], malaria screening [314,315,316,317,318], analysis of microcapillary occlusionsickle cell disease [319,320,321,322], sickle cell disease [323,324], detection and labeling of lymphoma cells [325], blood grouping and phenotyping [326,327], and the study of dispersive RBCs flowing through microchannels [328].

**Figure 16:**
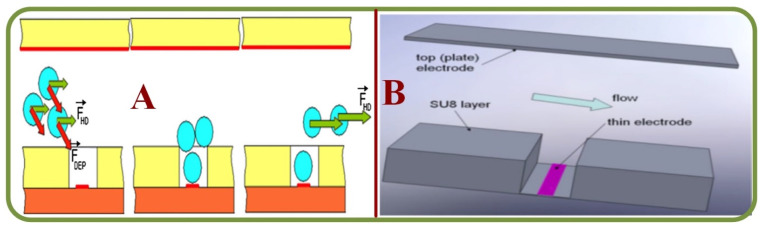
Examples of single RBC trapping arrays (**A**) A trapping device and cell patterning method using eDEP and iDEP [329]. (**B**) A single cell patterning method by using a strong DEP force, and removing unwanted cells by the hydrodynamic force [329].

Figure 16 shows some recent advancements in single RBC trapping arrays. Figure 16A shows the device structure and cell patterning method using eDEP and iDEP [329]. Figure 16B illustrates the single cell patterning method to trap cells using a strong DEP force, and to remove unwarranted cells by the hydrodynamic force [329].

Other examples of the microfluidic traps include a high-efficiency single RBC trapping scheme with integrated droplet generator [330], a microarray chip with trapped RBCs form a monolayer [331], an integrated Holographic Microscopy with single cell trapping and manipulating setup [332], single RBCs traps with label-free analyzer using high frequency ultrasound microbeams [333], and an all-fiber setup for the optical trapping of single RBCs [334]. In short, the advancements and potential future trends of Microarray-based single RBC manipulation techniques are summarized in the Table 4.

### 6.2. State of the Art in single Erythrocyte Trapping Techniques

Research has been significantly progressing in single-cell analysis with RBC/Erythrocyte trapping techniques. For example, diagnostic microarrays that can analyze protein samples via antibody-based microarrays have been shown as effective, time-saving, and label-free techniques for screening the RBC surface antigens and RBC phenotyping [335,336]. Sero-diagnostics from donor blood samples also holds potential for blood phenotyping, pathogenic infections, and blood typing from erythrocytes [337]. Microarray-based methods are consistent and reliable for genotyping applications of red blood cells and other blood components [338]. Furthermore, nickel mesh filters have been shown to be effective for the trapping of erythrocytes in suspensions [339]. On a similar note, optical trapping of erythrocytes using micropipettes embedded in a microfluidic system by the UV-Visible spectrophotometer is an excellent option for the analysis of various oxygenated states of RBCs [340]. In another work, a single beam acoustic trapping approach shows the potential for the assessment of interactive forces in RBCs [341]. Furthermore, it is found that the exposure of sickle red blood cells to epinephrine significantly increases the trapping of sickle cells and normal RBCs [342]. A highly sensitive device made of a polystyrene-based microarray has been fabricated to detect plasmodium infected RBCs utilizing a fluorescent detector [331,343]. An integrated optical system comprising 3-dimensional microscopy systems has been embedded for the trapping of RBCs to observe morphological changes [332]. For qualitative images, hypercalcemia has also been analyzed using an optical system embedded with optical tweezers with holographic microscopy [344].

### 6.3. Single RBC Trapping Forces

To facilitate and effectively study single-cell trapping, several actuating forces have been utilized in various setups based on both contact and non-contact schemes. This section briefly describes techniques that are utilized for the effective trapping of single cells.

#### 6.3.1. Acoustic Trapping

Acoustic tweezers can efficiently measure cell physiology and cell properties such as size, stiffness, and backscattering coefficients. These devices employ acoustic microbeams, which are originated from tightly focused high-frequency ultrasonic transducers. However, minor problems occur due to the instability of cells from instrumentation errors, leading to inaccurate measurements or cell ruptures due to uncontrolled acoustic pressures. Short ultrasounds with higher pulse repetition rates could be utilized to mitigate such issues. This method not only captures the entity of interest but is successful in elaborating its physical properties [333]. Studies have also indicated the prospects of analyzing cellular elasticity and viscous parameters for high-throughput applications to monitor diseases [345] and asses and induce deformation of RBC by ultrasonic standing wave [346]. Finally, there is the potential for single-beam acoustics in trapping RBCs from mixed suspensions. Moreover, nanoparticles can further facilitate the trapping procedure with externally applied electric potentials for in-vivo applications [347].

#### 6.3.2. Dielectrophoretic Trapping

Label-free dielectrophoresis methods can effectively assess cell biomechanics and biophysics. This method provides quantitative mechanical dispositions as a function of electro-deformation rates [348] as well as the prospects of cell capturing. Electro-deformation studies of single RBCs indicate a constant extensional recovery time for cell membranes [143]. Unconventional experiments of an insulator geometry with the DC field for trapping RBCs from human blood samples have exemplified that the capturing zones are generally regions with high dielectrophoretic forces [349]. Finally, a combined application of latex beads and erythrocytes using negative dielectrophoresis and hyper-layer field has shown that at higher frequencies, the erythrocytes are attracted towards the electrode with positive dielectrophoresis to be efficiently separated from the latex beads [350].

#### 6.3.3. Hydrodynamic Trapping

Hydrodynamic trapping utilizes mechanical barriers or arrays for the separation of target particles from the main flow and separated particles are retained within the hydrodynamic traps for further analyses. These traps are either contact-based or contactless and employ differential principles, such as cross streamed (viscoelastic focusing, inertial migration, dean flow and deformability selective cell separation), vortices based trapping (centrifugation assisted, cavitation microstreaming, hydrodynamic tweezers), and external controlled approaches (pneumatic valves, PID controllers, eddy currents, electro-magnetic fields, acoustics) [308]. Dielectrophoresis combined with hydrodynamic trapping has also been proposed for efficient cellular trapping, controlled contact between cells and objects and analysis [329,351]. Similarly, hydrodynamic and direct current-insulator-based dielectrophoresis have been tested for blood-plasma separation, along with observed trapping of RBCs [311]. A trap-and-release integrated system for dynamic microarray-based applications has also been proposed utilizing the hydrodynamic confinement and optical microbubbles for multiple bioanalytical applications [352]. The newly applied genedartive design method to hudrodynamic single RBC trap design showed highly promising. Though the number of cases with AI taking lead role in MEMS design is growing rapidly microfluidics has limited number of these interdisciplinary publications. Grigorev et al. applied Evolutionary approach for hydrodynamic traps design and achieved 4 out of 4 trapping efficiency of RBCs (within one FOV) in the fabricated and tested chip, after 30,000 solutions search. The optimized geometry was found to increase the through-slit velocities by 49% [353]. AI applied to the microfluidics design problems remains to be one of the potential growth areas in the near future [354].

#### 6.3.4. Magnetic Trapping

The existence of unpaired electrons within the heme group in human erythrocytes induces its paramagnetic properties, which is opposed to the diamagnetic behavior of oxyhaemoglobins. This enables the assessment of RBC migration based on magnetic characteristics [355]. RBCs can navigate in the solution using the magnetophoretic buoyancy and be efficiently trapped using this method due to its diamagnetic property [356]. Microfluidic constructs can also effectively trap and extract RBCs for application in blood phenotyping tests [296].

#### 6.3.5. Optical Trapping

Topics of RBC assessment using optical devices have been of immense interest, as they provide vital information regarding the microcirculatory parameters such as RBC aggregation forces using optical tweezers [357,358,359], and studies have pursued the assessment of aggregation dynamics [360]. One can actively implement this technology to assess biological parameters such as rheology and hemodynamics, although a few shortcomings related to effective measurements need reconsiderations [361]. While most of the experiments take place under oil-immersed objectives, further outlooks consider the prospects of water immersion objectives [361,362]. Waveguide-based optical traps also indicate good prospects. For example, waveguides fabricated from tantalum dioxide are efficient mediums for optical trapping and the propulsion of RBCs [363]. Studies related to the single RBC deformability in microchannels provide the recovery time for stretched RBCs, thereby furthering the prospects on RBC shape assessment [364]. Laser-based optical systems have been focused on the feasibilities of trapping platforms using femtosecond laser sources [334], with further implication in RBC imaging and assessment [365,366]. Chen et al. demonstrate the optical force controlled RBCs can act as bio-microlenses for trapping and imaging subwavelength objects [367]. Measuring erythrocyte deformability using laser-trapped methods has significant improved the efficiency and provided valuable information on RBC buckling, rigidity, and other viscoelastic properties [368,369,370,371]. Characterization of the trapping forces inside the optical manipulation mechanism can provide insights regarding the cellular-optical dynamics due to the trapping forces [372]. Another study demonstrates the duration of the post-optical tweezers exposure effect on RBC morphology and its membrane rigidness in adult blood [373] and umbilical cord blood [374]. Lastly, the aspects of single RBC ionization using laser-trapped techniques have also been considered, highlighting the potentials for measuring hemoglobin charges and other biophysical properties [375,376,377].

Raman spectroscopy plays a significant role in the observation and analysis of RBC dynamics inside trapped optical systems. Studies have focused on RBC responses to multiple situations, such as dextrose-containing intravenous fluids [378], the oxygenation cycle of trapped RBCs [379], Raman-based pH sensor for laser-trapped erythrocytes [380], and the study of eryptosis [381].

### 6.4. Limitations and Future Considerations for Single RBC Trapping Platforms

Although microarrays offer promising advancements in diagnostic testing and micromanipulation techniques, there are still large gaps in knowledge and prospects. The very first complication is the performance of cells on microarray surfaces, or the prospects for the enhancement of material surface interactions [335]. Advances in miniaturized and instantaneous Point-Of-Care (POC) systems are in desperate needs [336]. Assaying and identifying multiple blood types within a single device is an effective and time-saving method for analyses, and this could be included in the list of future developments [337]. Enhancing erythrocyte-trapping rates is also a vital consideration and can provide essential information related to hemorheological assessments [339]. Integration of spectroscopic facilities can also help developing advanced Lab-on-chip platforms for cell sorting and pharmaceutical applications. Last but not least, acoustic verification of cells can provide valuable information regarding intracellular biophysics [341]. The advancements and potential future trends of single RBC trapping techniques are summarized in the Table 5.

## 7. Organ-on-Chips, Multi-Organ Chips and Drug Discovery Involving Single RBC

### 7.1. Organ-on-Chips: State of the Art

In the view of ongoing research and development on tissue engineering, the combination of microfluidics and nanotechnology can help to close the gap between in-vitro and animal model-based studies. Organ-on-Chip (OOC) platforms can effectively reproduce the physiological traits of an organ within a microfluidic construct, e.g., spleen [382]. While these chips are mainly fabricated on PDMS, inherent complexities arise during its fabrication and require extra steps to alleviate the same. The combination of microfluidics and 3D printing for organ-on-chip applications is efficient for fabricating complex flow channels and provides the feasibility of creating biological structures with 3D cell distributions [383,384,385,386,387].

OOC-based studies have spread over multiple areas of microfluidic applications, including toxicity/drug testing and functional analysis of organs such as liver or spleen, lungs, intestine, kidneys, heart, vascular system, bone marrow, cancer/tumor cells, brain, and several others [388,389,390,391,392,393,394,395].

These studies have facilitated the visualization of cell and tissue physiology externally, including drug discovery and screening applications by allowing the repetitive, quantitative, and efficient study of drug interactions [396]. From the perspective of immunological analysis, OOC systems offer significant improvements over conventional methods, i.e., 2D-static culturing and animal model-based analysis. OOC also could be very useful in the areas of cultivating micro-pathophysiology for human immune systems [397].

Of all applications, the most common studies related to OOC have been on mimicking the human spleen and splenic filtration. The spleen is a secondary lymphoid organ responsible for the filtration of the infected and damaged RBCs via efficient networking and blood microcirculation through filtration beds, thereby allowing the recognition and destruction of unhealthy RBCs via microphages while keeping the healthy ones flowing. Researchers are attempting to replicate the splenic system to comprehend this filtration process. Initial considerations have included the hydrodynamic forces and the physical properties of the spleen along with its filtration capabilities [398,399]. RBC based microfluidic systems have been assessed for channel flow dynamics through biomimetic slits [400], internal channel hydrodynamics [401,402,403,404], liver-intestine and liver-skin culturing [405], splenic filtration of malaria-infected RBCs [406], dynamics inside alveolar capillaries [407], the culturing of multiple organs within a single chip [408], and the effects of hematopoiesis and counter-radiation responses in bone-marrows [409].

**Figure 17:**
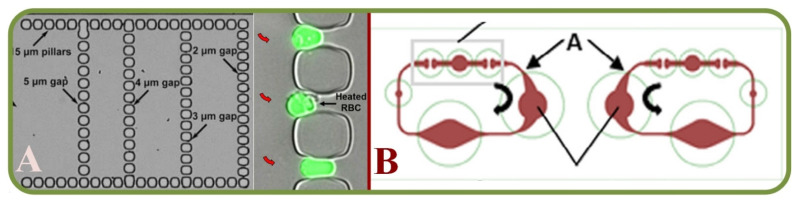
Organ-On-Chips and drug discovery involving single RBCs. (**A**) A biomimetic microfluidic chip to study the circulation and mechanical retention of RBCs in the spleen, including the top view of the fabricated filtration unit and the preferential retention of poorly deformable RBCs in slits [401]. (**B**) Chip-based human liver–intestine and liver–skin, including RBCs assisted microparticle imaging velocimetry setup and fluid flow circuits [405].

The National Institute of Health (NIH), the Food and Drug Administration (FDA), and the Defense Advanced Research Projects Agency (DARPA) have all made significant contributions to the OOC research and development. However, several key challenges in organ integration remain unresolved, including the disease modeling, individualized precision medicine, and clinical trials-on-chips for therapeutical research [410]. Figure 17 illustrates the recent advances in Organ-On-Chips and Drug Discovery involving single RBCs. For example, Figure 17A is a biomimetic microfluidic chip for studying the circulation and mechanical retention of RBCs in the spleen, including the top view of the fabricated filtration unit and the preferential retention of poorly deformable RBCs in slits [401]. A chip-based human liver–intestine and liver–skin and RBCs assisted microparticle imaging velocimetry setup and fluid flow circuits are shown in Figure 17B [405].

Furthermore, a 3D model of the four-organ-chip for interconnected co-culture of human intestine, liver, skin, and kidney equivalents was designed for RBC-based microparticle image velocimetry [408]. Another study covered the flow of RBCs through a narrow spleen-like slit and the visualization of cells under different viscosity ratios [404]. Yet another liver-on-a-chip platform, combined bio-printing of hepatic spheroids with bioreactor culture of hepatic constructs for biomarker analysis [411].

Other novel chips for the study of RBCs dynamics include the investigation of the human lungs and their terminal units (alveolar sacs) where pulmonary capillary network has lengths and diameters corresponding to the size of single RBCs [407]. Splenic filtration of Plasmodium falciparum-infected RBCs in malaria patients has been demonstrated to estimate the physical and fluorescent surface areas and the volume plots of normal and parasitized RBC distributions [406]. Another splenic study has analyzed the biomechanics of RBCs in human spleen for physiology and disease [402]. Splenic functions have also been studied with the functional model of the human splenon-on-a-chip mimicking filtering of healthy and unhealthy RBCs [398]. Maskless fabrication of microfluidic chips with localized surface functionalization for co-culture of cancer cells has facilitated the study of cell printing process with different printing heads such as the photopolymer head, UV head, and microplasma head [412].

### 7.2. Antibody Binding and Drug Discovery Applications

Pharmaceutical analytics is steadily gaining grounds in the area of drug development towards the implementations in drug analysis and drug screening, functional testing, and studies on metabolism. By embedding microfluidic chips with multiple detection modalities, high throughput screening, detection, and analysis of drug activities could be possible [413]. These techniques have been widely implemented to analyze blood typing and the screening of diseases through the interaction of antibodies with whole blood cells that initiate agglutination, which blocks the microflow network due to RBC depositions [414].

RBCs/erythrocytes have the potential as drug and nanoparticle carriers and are prospects for drug delivery methods employing internal and surface loading principles. Investigations have been trying to explore the applications of nanoparticles in RBCs, including prolonged circulation and stealth features as possible antigen carriers in immune response modulation [415].

Characterizing cell populations or immunophenotyping is a prevalent method for disease diagnosis from surface biomarkers using microfluidic arrays for label-free phenotyping [416] and interaction between RBC Glycophorin and leukocyte surface lectins activation [417].Other studies related to drug delivery assess the interaction mapping between RBCs and drug carriers via image analysis [418], capture and release of circulating tumor cells [419], drug-induced erythrocyte deformation [420], antibodies and biotin-labeled RBC effect on posttransfusion survival [421], shear-induced encapsulation in drug delivery [422], and anti-tumor applications incorporating RBCs and nanoparticles [423]. For COVID-19 related research, RBCs, as oxygen carriers, were analyzed for respiratory issues in COVID-19 patients for RBC deformability/morphology effect on tissues oxygen supply [424,425,426,427,428].

## 8. Future Considerations

The utilization of microfluidic chips for single RBC studies has been one of the most common analytical techniques due to unique capabilities in: (1) using only small amount of samples; (2) processing in a short period of time; (3) providing features such as integration/multiplexing; (4) suitable for automation for high-throughput and low-cost; (5) potentially disposable and portable [429]. Researchers in this field have highlighted the vast potential in developing micro devices for industrial and academia usages, such as various demonstration examples for lab-on-a-chip and organ-on-a-chip systems. This article has critically reviewed the current state-of-the-art and recent advances of microfluidics for single RBC analyses, including integrated sensing and microfluidic platforms for microscopic/tomographic/spectroscopic single RBC analyses, trapping arrays (including bifurcating channels), dielectrophoretic and agglutination/aggregation studies, as well as clinical implications covering cancer, sepsis, prenatal, and Sickle Cell diseases. Microfluidics based RBC microarrays, sorting/counting and trapping techniques (including acoustic, dielectrophoretic, hydrodynamic, magnetic, and optical techniques) have also been reviewed. Finally, organs on chips, multi-organ chips, and drug discovery involving single RBC have been introduced. The limitations and drawbacks of each technology have been analyzed and future prospects have been discussed.

The first important issue in terms of scientific, technological, and/or analytical matters is the methodology leading to the effective fabrication of targeted devices. The current state of the art studies have focused on laminates, molding-based, 3D printing, and micro/nano fabrication methods with distinct benefits and limitations [430,431,432]. Nevertheless, the primary goal of microfluidic single RBC research remains in finding the most convenient ways to advance biological and analytical studies in application-specific systems, such as chemotaxic assays, low resource diagnosis, and rapid assessment of bio-fluids [433]. These microfluidics chips have been implemented and studied globally to gain performance factors [434] for Point-of-Care (POC) diagnosis applications [435] and cellular studies [436,437,438]. There is also a demand for non-destructive and label-free analytical techniques for the rapid detection of erythrocytes’ pathologies and alterations on the molecular level, where Raman spectroscopy plays an important role [439]. Operational characteristics and the suitability for implementation in diagnosis have also been considered. On the other spectrum, there is clearly a strong need for POC devices in the developing countries. The manufacturing constraints [434] and material selections [430] play important roles in the development of single erythrocyte devices [436].

There are several other important directions for single RBC studies using microfluidic platforms. First, the integration of machine learning and deep learning algorithms for single RBC microfluidic systems has been becoming popular for improved performances in areas such as structural designs, signal analyses, and operational schemes. Second, further integrations to include more components in the system bears fruits of improvements in terms of different performance evaluation parameters, overall effectiveness, accuracy, and commercial suitability [440,441,442]. Third, RBC-based drug delivery system is another interesting track, where the cargo drug can be stored in the inner space enclosed by the plasma membrane and the outer surface of this membrane can feature unique and favorable pharmacokinetic and biodistribution characteristics [443].

Finally, efficient and high throughput systems are vital for the effective diagnosis and analysis of biological and internal pathophysiology. Several methodologies have been classified based on various factors and separation schemes [265]. In general, more sophisticated and integrated platforms are desirable to efficiently handle these complex analyses. There are also applications in the molecular diagnostics with good prospects toward commercialization [444], including microscopy-based analysis of RBCs [445], image-based RBC motion tracking [446], detection of thrombosis and hemostasis [447,448], immune checkpoint therapies [449], and the “liquid biopsy” of circulating tumor cells and fetal nucleated RBCs [450].

## Figures and Tables

**Table 1:** Comparison of Existing studies and Future directions.

Methodology	Existing Knowledge	Current Limitations	Future Prospects
Microscopic RBC/ Erythrocyte analysis	Viscosity aggregation of blood through T-junctions [25], RBC Quantitative Phase Imaging (QPI) [26,28] Quantitative Phase Microscopy (QPM) [29,30] Microvasculature on a chip [27], light scattering [31], saline induced stiffness [43]	Relationships between RBC aggregation and blood viscosity; refocusing issues in phase imaging systems and spatiotemporal phase sensitivity.	Viscosity in complex networks, phase imaging cytometry, cell aggregation, waveguide-based microfluidic platforms, multicellular diagnosis.
Spectral Imaging	State-of-the-art [58], RBC physics and mechanics [59,61], POC malaria diagnosis [60], aggregation dynamics [63], hybrid analytical platforms [62]	Low processing speeds, limitations in wavelength and challenges due to the variation of device elements.	Multispectral disease diagnosis, spectral cellular deformability analysis, spectral POC systems.
Raman Spectra Applications	State-of-the-art [64], isolation of circulating cells [65], clinical diagnosis [66,67,68], bio-particle trapping [69], blood plasma [70], SERS RBC analysis [73,74], optical-guided cell focusing [75], Hb oxygenation uptake [77]	Raman scattering related issues, signal overlap, long exposure time, channel dimension and optical scattering trade-off, integrating SERS systems and dynamic trapping of cells, bulky components.	Precision diagnostics for cancer metastasis, advanced laser-embedded microfluidic chips, multi-channel Raman analytics and sorting, multiplexing of analytes, real time analytics, reusability.
Tomographic Analysis	3D microchannel tomography [46], OCT angiography [47], label-free cytometry [48,50], phase-contrast tomography (PCT) [49], hyperspectral tomography [51]	Flow cytometry relies on 1D assessment, limited wavelength, false-positive signals, acquisition of flow signal intensity, limited field of view and acquisition time.	Shape-based cellular identification, fast acquisition speed, optimal wavelengths, motion correction, detection of circulating tumour cells, 3D image reconstruction.
Miscellaneous Studies	Waveguide based blood typing [79], optical tweezers [80], speckle analysis [81,82,84], machine vision analytics [83]	Equipment handling, uncertainties in the relationship between parameters, scatter concentrations affect speckle contrast	Stability and accuracy in agglutination detection, stem cell-based therapy, moving trajectory-based deformation, speckle decorrelation time.

**Table 2:** Microfluidic devices for RBC Assessments in Clinical Practice.

Domain	State of the Art	Limitations	Future Prospects
General [197,198,199,200,201,202,203,204,213,214,215,216,217]	RBC Sedimentation, real-time tracking, finite element based analysis, POC systems, coagulation–aggregation, fibrinogen-RBC interactions, optical detections	Device reliability, component incompatibility, assess limits in immunological profiles, occlusions, quality control, the high surface area-volume ratio to modify adsorption-desorption characterics.	Detection of tumor cells, advanced data acquisitions, macromolecules for drug delivery, hydrodynamic focusing, smartphone integrated devices, reduced testing time, simultaneous assaying, microfluidic vascular models, precise fabrication of sub-components.
Malaria infected RBC/Erythrocytes [224,225,226,228,255]	Deformation analysis, paper-based POC devices, microarray and microchannel analytics, cell-phoresis analytics, impedance cytometry	Automated devices, high-performance reagents, integrated systems, detection limitations, false-positive cases, drug reactions	Centralized geographical tracking, drug discovery platform, anti-malarial therapy, detection of RBC deformability with label-free identification and sample pre-enrichment
Sickle Cell Disease [230,231,232,233]	RBC adherence and cell phenotyping, hemoglobin content and hemolysis, vaso-occlusion due to SCD, micro-particle image velocimetry	Custom design for RBC adhesion studies, a traditional dish based cell culturing, internal bubble growth, vaso-occlusive crisis	Hemoglobin, reticulocyte, and lactate dehydrogenase count, RBC adhesion biophysics and hemolysis, vaso occlusion on sickling kinetics, poloxamer for decreasing blood viscosity, hematological parameters, drug interaction study
Sepsis [234,235]	RBC deformation mechanics, neutrophil motility analysis, laser diffraction studies	Limited knowledge on the impacts of neutrophils in the cytokine promotion	The correlation between neutrophils and septic responses, neutrophil activation, identification of inflammatory sequences, and erythrocyte rigidity
Cancer [237,238,239,241,242]	Droplet-Based ScRNAseq, acoustic cell separation, micro-filters and dielectrophoresis, magnetic cell navigation for photodynamic therapy, image-guided therapies	Lymphocyte contamination, cell concentration, processing rate dependence during sorting, loss of residual samples	Post-separation cell culturing, ex-vivo drug screening, anticancer drug accumulation, multidrug resistance, targeted drug delivery, synthesis and storage of theranostic nanoparticles, microfluidic electroporation, cell membrane coated nanoparticles
Prenatal Diagnostics [243,244,245]	Fetal RBC collection, loss of diseased RBC, magnetic enrichment, and nucleated RBC isolation	Scarcity of fetal cells in maternal circulation, high priced detection equipment	Rare cell separation, advanced molecular biology analytics, capture of limited fetal cells, microarray-based detections, circulating tumor cells for genetic tests
Miscellaneous [207,209,211,212,246,247,248,249,250,251,252,253,256]	Numerical and computational cell biomechanics, anemia detection, blood vessel interactions, drug interaction models, optical tweezers-based devices, silica-monolith platforms, spectroscopic techniques.	Interaction of blood cells under varying flows, metabolism-related alterations, immuno-suppression due to allogenic RBC transfusion, limited sampling volume, lack of knowledge on cell flow behavior, manufacturing cost	RBC biomechanics in metabolic disorders and diabetic neuropathy, construction of kinetic-particle models, POC based hemoglobin tests, RBC transfusion induced vascular injuries, assessment of protein-concentration effects, nanofluidic filtration and spectroscopic coupling, RBC micro motor based theranostics, blood coagulation and platelet defects

**Table 3:** Microfluidic Single-RBC Filtering Techniques [257,262,271].

Method	Advantages	Disadvantages
Physical filtration	High separation and sorting efficiency	Clogging and fouling of cells
Hydrodynamic and hemodynamic processes	Enhanced separation and sorting with narrowed sheath flows	Stress on cells, altering molecular mechanisms, inhomogeneity
Surface Affinity and Topography	Specificity and accuracy	Altering cell physiology
Magnetophoresis	Differentiating cells without additives, efficiency up to 90%	Magnetic flux gradients on cells
Electrical Methods and Acoustophoresis	Sensitive, rapid, convenient, and robust.	Electrolysis, temperature elevations, phenotypic changes

**Table 4:** Microarray-based Single RBC Manipulation Techniques.

Technique	State of the Art	Future Prospects
**Immuno-phenotyping applications** [335,336,337,338]	Detection of complex mixtures and antigens, pre-transfusion testing, serodiagnosis, and genotyping	Futuristic assaying methodology, device miniaturization, and combined testing
**Mesh filtration and Erythrocyte deformability analysis** [339]	RBC rheology through a nickel mesh filter	Further studies of trapping rates and rheology
**Trapping Spectroscopy** [340]	Micropipette based multifunctional microfluidic trapping	Cell sorting, pharmaceutics, Point of Care testing
**UHF Single Beam acoustic tweezers** [341]	Effective measurement of inter RBC forces	Other types of cells
**Sickle Cell adhesion** [342]	Sickle cell adhesion analysis, vaso occlusion studies	Development of anti-adhesive/occlusion agents, stress-dependent vaso-occlusion studies
**Malaria Detection** [331,343]	Highly sensitive detection of infection rates, remote analysis	Large-scale and mobile diagnostics
Digital Holographic Microscopy [332,344]	Optical trapping and recording via digital holograms	Detailed cellular dynamics, morphology-based identification

**Table 5:** Single RBC trapping techniques.

Trapping	State of the Art	Current Limitations	Future Prospects
Acoustic	Label-free approach for the analysis of physical properties [333], elastic and viscous parameters [345], trapping under suspensions [347]	Isolation and characterization of single cells, high-intensity ultrasound for cell trapping, increase in local temperature, acoustic rupture	Relation between particle diameter and ultrasound wavelength for trapping, automated analysis of erythrocyte population, in vivo application of single beam tweezers
Dielectrophoretic	single cell deformation mechanics [143,348], insulator based gradient dielectrophoretics [349], erythrocyte separation [350]	Particle sub-populations	Biophysical property determination, separation of bio analytes from complex fluids, treated surface coating, DC-micro devices, ellipsoid model-based RBC analysis
Hydro-dynamic	DC insulator-based [311], Steady stream flows, stagnation traps. hydrodynamic traps [308,329,352]	Cell retention vs. trapping efficiency trade-offs, complex fabrication processes, choking due to high cell density, loss of smaller cells due to non-uniformity	Enhanced particle (cell) separation, automated pumping systems, genetic/ biochemical/ physiological cell studies, reduced mechanical shear on cells, biocompatible materials
Magnetic	Improved magnetic field gradients and counter-current flow trapping [356], magnetophoretic RBC migration analysis [355]	Low volume analysis, pre-treatment methods, pre-labeling	Oxygenated erythrocyte mobility analysis, paramagnetic behavior studies, superconductive fractionation, collection throughput, low cost, rapid and automation
Optical	Cell aggregation [357,358,359], objective immersion-based [362], deformability analysis [361], waveguide surface-based [363]	Optical assessment of RBC mechanics, spontaneous aggregation modeling, stiffness calibration	Improved RBC shape recovery, cell positioning, aggregation rate, cell sorting, drug delivery

## Data Availability

No new data was created or analyzed in this study. Data sharing is not applicable to this article.
